# Associations of Bariatric Interventions With Micronutrient and Endocrine Disturbances

**DOI:** 10.1001/jamanetworkopen.2020.5123

**Published:** 2020-06-09

**Authors:** Nicholas L. Syn, Phong Ching Lee, Jean-Paul Kovalik, Kwang Wei Tham, Hock Soo Ong, Weng Hoong Chan, Chuen Seng Tan, Chin Hong Lim

**Affiliations:** 1 Department of Upper Gastrointestinal and Bariatric Surgery, Singapore General Hospital, Singapore; 2 Yong Loo Lin School of Medicine, National University of Singapore, Singapore; 3 Department of Endocrinology, Division of Medicine, Singapore General Hospital, Singapore; 4 Cardiovascular and Metabolic Diseases Programme, Duke-NUS Medical School, Singapore; 5 Biostatistics and Modelling Domain, Saw Swee Hock School of Public Health, National University Health System, Singapore

## Abstract

**Importance:**

Few studies have described the longitudinal trajectories of serum levels of micronutrients whose deficiencies are associated with serious sequelae following bariatric procedures, such as anemia, osteoporotic fractures, and neuropathies. Furthermore, previous studies comparing laparoscopic sleeve gastrectomy (LSG) vs Roux-en-Y gastric bypass (LRYGB) or one-anastomosis gastric bypass (OAGB) procedures may have been limited by selection and confounding biases.

**Objective:**

To appraise the spectrum and temporal course of micronutrient deficiencies associated with bone metabolism and erythropoiesis after LSG vs OAGB or LRYGB procedures, using the propensity score as a balancing score.

**Design, Setting, and Participants:**

This prospective, longitudinal comparative effectiveness study was conducted at a high-volume bariatric unit in Singapore from September 1, 2008, to November 30, 2017, with a cutoff date for analysis of September 2018. Patients who underwent adjustable gastric banding, biliopancreatic diversion procedures, and intragastric balloon procedures were excluded. All other patients who underwent bariatric procedures were included. Data were analyzed from September 23 to 30, 2018.

**Main Outcomes and Measures:**

Serial assessment of 13 biochemical parameters at 12 time points for up to 5 years after bariatric procedure. Inverse probability-of-treatment weights were used to obtain estimates of the mean associations of variables assessed with the bariatric surgical interventions. Longitudinal trajectories were analyzed using mixed-effects generalized linear models to apportion the temporal variation of serum micronutrients into fixed-effects and random-effects components.

**Results:**

A total of 688 patients were included in this study, of whom 499 underwent LSG (mean [SD] age, 41.5 [11.3] years; 318 [63.7%] women) and 189 underwent OAGB or LRYGB (mean [SD] age, 48.6 [9.4] years; 112 [59.3%] women). There were no differences during follow-up among patients who underwent LSG vs those who underwent OAGB or LRYGB in intact parathyroid hormone levels (mean difference, 7.05 [95% CI, −28.67 to 42.77] pg/mL; *P* = .70), serum 25-hydroxyvitamin D levels (mean difference, −0.72 [95% CI, −1.56 to 0.12] ng/mL; *P* = .09), or phosphate levels (mean difference, 0.006 [95% CI, −0.052 to 0.064] mg/dL; *P* = .83). Hemoglobin levels were a mean 0.63 (95% CI, 0.41 to 0.85) g/dL higher among patients who underwent LSG compared with those who underwent OAGB or LRYGB (*P* < .001), despite no differences in iron concentration levels (mean difference, 1.50 [95% CI, −1.39 to 4.39] µg/dL; *P* = .31), total iron-binding capacity (mean difference, 4.36 [95% CI, −5.25 to 13.98] µg/dL; *P* = .37), or ferritin levels (mean difference, 3.0 [95% CI, −13.0 to 18.9] ng/mL; *P* = .71). Compared with patients who underwent LSG procedures, patients who underwent OAGB or LRYGB had higher folate levels (mean difference, 2.376 [95% CI, 1.716 to 3.036] ng/mL; *P* < .001) but lower serum magnesium levels (mean difference, −0.25 [95% CI, −0.35 to −0.16] mg/dL; *P* < .001) and zinc levels (mean difference, −7.58 [95% CI, −9.92 to −5.24] µg/dL; *P* < .001).

**Conclusions and Relevance:**

These findings suggest that LSG vs OAGB or LRYGB procedures have differential associations with various micronutrient and metabolic parameters. These differences should be recognized in guidelines for postbariatric nutritional surveillance and prevention.

## Key Points

**Question:**

Are bariatric interventions associated with serum micronutrients and metabolic hormones changes in the years after intervention, and do trajectories differ between laparoscopic sleeve gastrectomy vs gastric bypass procedures?

**Findings:**

In this comparative effectiveness study of 499 patients who underwent sleeve gastrectomy and 189 patients who underwent one-anastomosis or Roux-en-Y gastric bypass, parameters associated with calcium metabolism did not differ between the groups. The extent of hemoglobin suppression was greater among patients who underwent gastric bypass compared with those who underwent laparoscopic sleeve gastrectomy, but the difference was not explained by differences in iron stores or vitamin B levels.

**Meaning:**

The findings of this study suggest that micronutrient deficiencies after bariatric interventions are differentially associated with types of bariatric procedures.

## Teaser

This comparative effectiveness study examines the association of bariatric interventions with micronutrient deficiencies and endocrine disturbances.

## Introduction

While effective at inducing substantial and durable weight loss, bariatric procedures are recognized to predispose patients to vitamin and mineral deficiencies.^1,2,3,4^ The prevailing notion is that the extent and spectrum of these deficits are contingent on the physiological and anatomical alterations imposed by the different types of bariatric procedures.^5,6,7,8,9,10,11^ Procedures that achieve weight loss predominantly through restriction of meal accommodation capacity (known as *restrictive procedures*) are thought to be associated with milder micronutrient deficiencies that occur in tandem with reduced food intake. In contrast, interventions with a malabsorptive component (known as *malabsorptive procedures*) are thought to be associated with more profound and focal nutritional deficits and metabolic derangements, as they entail bypassing alimentary tract segments involved in the absorption of specific dietary nutrients. For example, the duodenum and proximal jejunum serve as critical absorption sites for trace elements, such as zinc, copper, and calcium,^1,2^ and also contain free fatty-acid receptors that modulate cholecystokinin secretion and hence affect absorption of fat-soluble vitamins (eg, A, D, E, and K).^12^

Long-term micronutrient deficiencies are likely associated with the elevated incidence of osteoporotic fractures, secondary hyperparathyroidism, anemias, and other late sequelae of bariatric procedures.^12,13,14,15,16,17,18,19^ However, the longitudinal trajectories of serum micronutrient levels remain unclear because most previous studies have analyzed data at single cross-sectional points (eg, at 1-year or 3-year follow-up), which may not be representative of temporal fluctuations of micronutrient levels over time. Thus, the aim of this study was to delineate a clearer picture of the extent and severity of micronutrient deficiencies associated with laparoscopic sleeve gastrectomy (LSG) compared with laparoscopic Roux-en-Y gastric bypass (LRYGB) or one-anastomosis gastric bypass (OAGB) using longitudinal mixed-effects models.^20,21^ To minimize confounding and selection biases, analyses were adjusted using inverse probability-of-treatment weights (IPTWs) based on propensity scores.^22,23,24,25,26,27,28,29^

## Methods

Ethical approval to conduct this study was granted by the SingHealth institutional review board with waiver of informed consent because data were deidentified. The final data set used for analyses was retrieved on September 23, 2018. To ensure relevance to stakeholders, we reviewed the International Society for Pharmacoeconomics and Outcomes Research (ISPOR) and Strengthening the Reporting of Observational Studies in Epidemiology nutritional epidemiology extension (STROBE-nut) reporting guidelines for comparative effectiveness research using nonrandomized cohort studies.

### Study Population, Interventions, and Outcome Assessments

Data for this observational comparative effectiveness study were obtained from a prospective bariatric procedure registry of consecutive patients maintained at the Singapore General Hospital, Singapore, from September 1, 2008, to November 30, 2017. Singapore is a multiracial country with a population that includes ethnic Chinese (76.2%), Malay (15.0%), and Indian (7.4%) individuals.^30^

Patients undergoing bariatric interventions at our institution are treated by a specialized, multidisciplinary bariatric unit comprising bariatric surgeons, endocrinologists, and registered dietitians. All LSGs were performed using 5 ports placed in the upper abdomen as previously described.^31^ The abdominal cavity was insufflated with carbon dioxide and intraabdominal pressure maintained at 15 mm Hg. Dissection commenced at approximately 3 cm proximal to the pylorus; the omentum was separated from the greater curvature by dividing the branches of the gastroepiploic vessels and the short gastric vessels using a Harmonic scalpel (Ethicon Endosurgery). Special attention was paid in completely exposing the left crus of the diaphragm and ensuring complete clearance of the posterior aspect of the fundus. A 120-mm calibration tube was inserted orally and the stomach tubularized with the application of an endoscopic stapler (Echelon-Flex green, gold, and blue cartridges, Ethicon Endosurgery). The disconnected stomach was removed in an endoscopic bag (Endo-Catch 15 mm, Medtronic) via a 15-mm opening at the umbilicus. For LRYGB procedures, a 20-mL lesser curve-based gastric pouch was fashioned over a 120-mm orogastric tube, with a 100-cm biliopancreatic limb and 100-cm antecolic Roux limb with stapled gastrojejunostomy. Mesenteric defects were closed.^32^

For the purpose of dichotomization, we classified procedures as being predominantly restrictive (ie, LSG) or predominantly malabsorptive (ie, LRYGB and OAGB). However, in reality, it should be acknowledged that many bariatric interventions incur weight loss through a mixture of restrictive and malabsorptive mechanisms. All patients were dispensed standardized dietary advice regarding staged meal progression and macronutrient proportions (eg, protein intake of at least 1 g per 1 kg of ideal body weight) under supervision of registered dietitians, consistent with clinical practice guideline recommendations.^33^ Per our institution’s standard operating practice, nutritional deficiencies identified prior to surgical procedures were orally replaced. After bariatric intervention, patients were prescribed the following supplements per our unit protocol: 1 to 2 multivitamin tablets daily, elemental calcium (1000-2000 mg per day), and vitamin D_2_ (50 000 IU 1 to 2 times per week). The multivitamin tablet in our study is a commercially available prenatal formulation that includes vitamin A (3000 IU), thiamine (10 mg), folic acid (1000 µg), copper (100 µg), and iron (ferrous fumarate, 30 mg). Subsequent doses of supplements were titrated according to 6 to 12 monthly routine laboratory measurements. Supplemental oral iron formulations, vitamin B_12_, and folic acid were not standardized or enforced but could be ordered at the treating physician’s discretion if patients were deemed at risk of developing anemia (eg, patients whose hemoglobin levels or other iron-related parameters appeared to be trendling lower over time, those with a sharp decline in hemoglobin levels between any 2 consecutive visits, or women reporting a history suggestive of menorrhagia) or if deficiencies were identified during follow-up. Ergocalciferol (vitamin D_2_), instead of cholecalciferol (vitamin D_3_), was the choice of vitamin D replacement in this study, as it was the only high-dose vitamin D formulation available at our institution. The high-dose vitamin D replacement regimen used at our institution is based on several endocrine society guidelines that have advocated aggressive vitamin D supplementation following a bariatric procedure.^33,34^ To ensure adequacy of vitamin D replacement without incurring toxic effects, 25-hydroxyvitamin D levels were closely monitored, and the dose of vitamin D_2_ was titrated to achieve a target 25-hydroxyvitamin D level of 30 ng/mL [to convert to nanomoles per liter, multiply by 2.496]. Patient adherence to all supplements was assessed and reinforced at each visit.

All laboratory tests were performed in the fasted state, and serum parameters included albumin, calcium, phosphate, intact parathyroid hormone, 25-hydroxyvitamin D, ferritin, iron, hemoglobin, total iron-binding capacity, vitamin B_12_ (ie, cobalamin), vitamin B_9_ (ie, folate), zinc, and magnesium. These assessments were performed at 12 predefined points (ie, 1, 3, 6, 9, 12, 18, 24, 30, 36, 48, and 60 months after bariatric intervention) for up to 5 years.

### Statistical Analysis

To estimate mean associations for the interventions and minimize selection and confounding biases, we performed a comparative effectiveness study using the propensity score as a balancing score. Variables were selected for inclusion into a penalized logistic regression model using a L1-regularized procedure based on the least absolute shrinkage and selection operator λ penalty, the optimal value of which was determined through 10-fold cross-validation to give the minimum cross-validated error. To enhance statistical power, baseline covariates included in the propensity score model as well as missing follow-up outcome data were multiply imputed (50 imputations) using multivariate chained equations with the following specifications: predictive mean matching for continuous variables (5 k–nearest neighbors), augmented logistic regression for binary variables, and ordinal logistic regression for ordered categorical variables (eg, normal, prediabetes, and diabetes status).^35,36,37^ Individuals with missing outcome data in the immediate postoperative period were excluded from the imputation procedure. Propensity scores for individual patients were then computed by combining the postestimation probabilities across 50 multiply imputed data sets per Rubin rules.

To efficiently use all longitudinally repeated measurements of micronutrient and hematological or endocrine parameters, generalized linear mixed models were carried out to apportion the temporal variation of serum micronutrients into fixed-effects and random-effects components.^35,36,37^ This was accomplished by incorporating a treatment-by-time interaction with an unstructured covariance and Huber-White robust variance estimators. The fixed-effects component can be interpreted to reflect the true underlying trajectory of the outcome parameter at the population level, while the random-effects terms are used to model interindividual and interoccasion variability. All mixed-effects analyses were adjusted using IPTW, and we additionally adjusted for sex in our models, as sex was not accounted for in the propensity score but could nevertheless represent a potential confounder because of physiological differences between the sexes. Comparisons at different points or between the 2 types of bariatric procedures were calculated using predictive margins or contrasts from the postestimation linear combinations of coefficients. Finally, we also repeated all analyses using the raw data (ie, without multiple imputation or IPTW), and the results of these sensitivity analyses are reported in the eAppendix in the Supplement.

Unadjusted comparisons of baseline characteristics were performed using unpaired *t* tests for continuous variables, Pearson χ^2^ test for categorical variables, and log-rank test for time lapse between diagnosis of diabetes to bariatric procedure. To assess whether there was residual statistical imbalance after conditioning baseline distributions on the propensity score, we repeated the comparisons with inverse probability weighted linear regressions for continuous variables, logistic regression for categorical variables, and Cox regressions time-to-event variables. All statistical analyses were performed in Stata statistical software version 16.0 (StataCorp). *P* values were 2-sided, and *P* < .05 was regarded to indicate nominal statistical significance.

## Results

### Baseline Patient Characteristics

In total, 688 patients were included, of whom 499 underwent LSG (mean [SD] age, 41.5 [11.3] years; 318 [63.7%] women) and 189 underwent OAGB or LRYGB (mean [SD] age, 48.6 [9.4] years; 112 [59.3%] women). The propensity-score model exhibited excellent discrimination (area under the curve, 0.9135 [bootstrapped 95% CI, 0.8891-0.9380]) and calibration (Hosmer-Lemeshow test *P* = .73) (eFigure 1 and eFigure 2 in the Supplement). Factors associated with assignment to LSG compared with OAGB or LRYGB included higher body mass index (odds ratio [OR], 1.14 [95% CI, 1.07-1.22]; *P* < .001), younger age (OR, 0.94 [95% CI, 0.90-0.98]; *P* = .004), and lower-risk glycemic status (prediabetes: OR, 0.34 [95% CI, 0.17-0.66]; *P* = .002; diabetes: OR, 0.07 [95% CI, 0.04-0.12]; *P* < .001) (Table). Additional demographic and clinical characteristics are summarized in the Table, and baseline covariates were well balanced after conditioning their distributions on the propensity score.

**Table.** Comparisons of Baseline Clinical and Demographic Characteristics Between Patients Undergoing LSG vs OAGB or LRYGB Bariatric Procedures, and Covariates Included in Propensity-Score Model

Characteristic	Patients, mean (SD)		Unadjusted *P* value from univariable comparisons	Adjusted *P* value from inverse probability-weighted comparisons^a^	Multivariable odds ratio (95% CI)	*P* value
	Undergoing LSG (n = 499)	Undergoing OAGB or LRYGB (n = 189)				
Age, y	41.5 (11.3)	48.6 (9.4)	.002	.60	0.94 (0.90-0.98)	.004
Men, No. (%)	181 (36.3)	77 (40.7)	.30	.98		
BMI	44.0 (6.9)	38.3 (7.1)	<.001	.55	1.14 (1.07-1.22)	<.001
Preoperative weight, kg	117.1 (24.9)	109.3 (22.9)	<.001	.45		
Fat mass, kg	63.3 (19.1)	50.9 (17.2)	<.001	.41	NA	NA
Free fat mass, kg	54.4 (16.8)	54.8 (13.4)	.89	.71	NA	NA
Race/ethnicity, No. (%)						
Chinese	187 (37.5)	83 (43.9)	.31	.72^b^	NA	NA
Malay	157 (31.5)	55 (29.1)			NA	NA
Indian	122 (24.5)	44 (23.3)			NA	NA
Other	33 (6.6)	7 (3.7)			NA	NA
Glycemic status, No./total (%)						
Within reference range	270/465 (58.1)	23/184 (12.5)	<.001	.21^c^	1 [Reference]	NA
Prediabetes	72/465 (15.5)	18/184 (9.8)			0.34 (0.17-0.66)	.002
Diabetes	123/465 (26.5)	143/184 (77.7)			0.07 (0.04-0.12)	<.001
Time since diagnosis, median (IQR), mo^d^	24 (8-90)	115 (36-180)	<.001	.18		

Abbreviations: BMI, body mass index (calculated as weight in kilograms divided by height in meters squared); IQR, interquartile range; LSG, laparoscopic sleeve gastrectomy; LRYGB, laparoscopic Roux-en-Y bypass; NA, not applicable; OAGB, one-anastomosis gastric bypass.

^a^ Adjusted *P* value from inverse probability-weighted comparisons (*P* > .05 indicates that distributions conditioned on the propensity-score are balanced).

^b^ Calculated from the likelihood ratio χ^2^ test that for the separate equations comprising a multinomial logit model (with inverse probability weights), at least one of the regression coefficients is not equal to zero.

^c^ Calculated from an ordinal logit model with inverse probability weights.

^d^ Calculated among patients with preexisting type 2 diabetes.

### Calcium Metabolism

**Figure 1.**
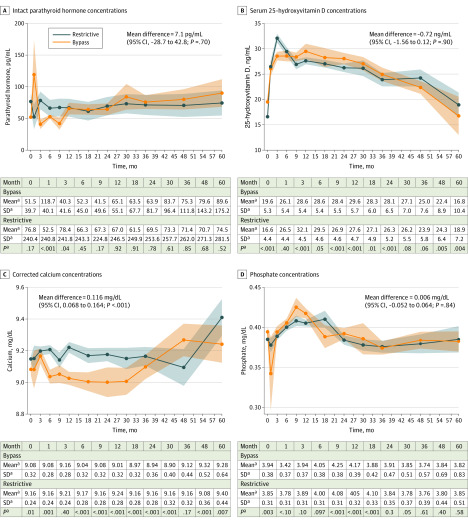
Five-year Trajectories in Serum Hormone and Micronutrient Concentrations SI conversion factors: To convert parathyroid hormone level to nanograms per liter, multiply by 1; 25-hydroxyvitamin D level to nanomoles per liter, multiply by 2.496; calcium to millimoles per liter, multiple by 0.25; and phosphate to millimoles per liter, multiply by 0.323. ^a^Estimated margins and 95% CIs are plotted immediately after fitting inverse probability of treatment weighted linear mixed models conditioned on the treatment propensity score and additionally adjusted for sex. Restrictive includes patients who underwent laparoscopic sleeve gastrectomy; bypass includes patients who underwent laparoscopic Roux-en-Y gastric bypass or one-anastomosis gastric bypass.

During follow-up, intact parathyroid hormone levels were not found to differ among patients who underwent LSG vs those who underwent OAGB or LRYGB (mean difference, 7.05 [95% CI, −28.67 to 42.77] pg/mL [to convert to nanograms per liter, multiply by 1]; *P* = .70) (Figure 1A; eFigure 3 in the Supplement). In the overall cohort, mean (SD) serum 25-hydroxyvitamin D concentrations were higher at all postoperative time points compared with baseline levels (baseline: 17.7 [7.3] ng/mL; 1 month: 26.9 [10.7] ng/mL; 3 months: 31.3 [11.2] ng/mL; 6 months: 29.5 [9.9] ng/mL; 9 months: 27.8 [8.0] ng/mL; 12 months: 29.2 [9.9] ng/mL; 18 months: 27.8 [8.9] ng/mL; 24 months: 28.1 [8.6] ng/mL; 30 months: 27.2 [9.9] ng/mL; 36 months: 27.7 [8.9] ng/mL; 48 months: 26.5 [8.0] ng/mL; 60 months: 25.7 [7.7] ng/mL; *P* < .001) (eAppendix and eFigure 3 in the Supplement). There was no overall difference in serum vitamin D levels between patients who underwent LSG compared with those who underwent OAGB or LRYGB during follow-up (mean difference, −0.72 [95% CI, −1.56 to 0.12] ng/mL; *P* = .09), despite the fact that vitamin D levels differed significantly between both groups at many of the individual follow-up points (Figure 1B). Compared with patients who underwent OAGB or LRYGB, patients who underwent LSG procedures had statistically higher corrected calcium levels (mean difference, 0.12 [95% CI, 0.07 to 0.16] mg/dL [to convert to millimoles per liter, multiple by 0.25]; *P* < .001), but not phosphate levels (mean difference, 0.006 [95% CI, −0.052 to 0.064] mg/dL [to convert to millimoles per liter, multiply by 0.323]; *P* = .83) (Figure 1C and D; eFigure 4 in the Supplement).

### Iron Metabolism

**Figure 2.**
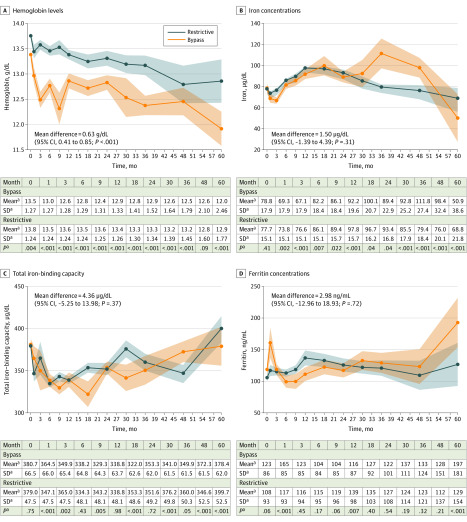
Five-Year Trajectories in Hemoglobin and Iron-related Serum Concentrations SI conversion factors: To convert hemoglobin to grams per liter, multiply by 10; iron to micromoles per liter, multiply by 0.179; and ferritin to micrograms per liter, multiply by 1. ^a^Estimated margins and 95% CIs are plotted immediately after fitting inverse probability of treatment weighted linear mixed models conditioned on the treatment propensity score and additionally adjusted for sex. Restrictive includes patients who underwent laparoscopic sleeve gastrectomy; bypass includes patients who underwent laparoscopic Roux-en-Y gastric bypass or one-anastomosis gastric bypass.

During 5 years of follow-up, hemoglobin levels were a mean of 0.63 (95% CI, 0.41 to 0.85) g/dL (to convert to grams per liter, multiply by 10) higher among patients who underwent LSG compared with those who underwent OAGB or LRYGB (*P* < .001) (Figure 2A). In both groups, hemoglobin concentrations decreased in a largely monotonic fashion, although patients who underwent OAGB or LRYGB recorded a steeper decrease in mean (SE) hemoglobin levels during the first 3 months compared with those undergoing LSG (OAGB or LRYGB: baseline: 13.4 [0.12] g/dL; 1 month: 13.1 [0.18] g/dL; 3 months: 12.7 [0.14] g/dL; LSG: baseline: 13.8 [0.08] g/dL; 1 month: 13.5 [0.14] g/dL; 3 months: 13.6 [0.10] g/dL). Hemoglobin trough levels were recorded at 48 months after bariatric procedure (LSG: −0.8 [95% CI, −1.3 to −0.4] g/dL; *P* < .001; OAGB or LRYGB: −0.9 [95% CI, −1.3 to −0.6] g/dL; *P* < .001) compared with levels measured prior to bariatric intervention (eAppendix, eFigure 5, and eFigure 6 in the Supplement). However, there were no differences between patients who underwent LSD compared with those who underwent OAGB or LRYGB in iron concentration (mean difference, 1.50 [95% CI, −1.39 to 4.39] µg/dL [to convert to micromoles per liter, multiply by 0.179]; *P* = .31), total iron-binding capacity (mean difference, 4.36 [95% CI, −5.25 to 13.98] µg/dL; *P* = .37), or ferritin levels (mean difference, 3.0 [95% CI, −13.0 to 18.9] ng/mL [to convert to micrograms per liter, multiply by 1]; *P* = .71) (Figure 2B-D; eFigure 7 and eFigure 8 in the Supplement).

Interestingly, in stratified analyses by sex, we detected a strongly monotonic, decreasing trend in hemoglobin levels among women (*P* < .001 for monotonicity) but not men (*P* = .78 for monotonicity) during follow-up (eFigure 6 in the Supplement). Furthermore, ferritin levels were consistently higher among women who underwent OAGB or LRYGB compared with LSG (mean difference, 31.1 [95% CI, 11.6 to 50.7] ng/mL; *P* = .002). Additional results concerning the differential associations of OAGB or LRYGB and LSG with iron-related parameters in both sexes are detailed in the eAppendix, eFigure 6, and eFigure 8 in the Supplement.

### Vitamin B Metabolism, Magnesium, and Zinc

**Figure 3.**
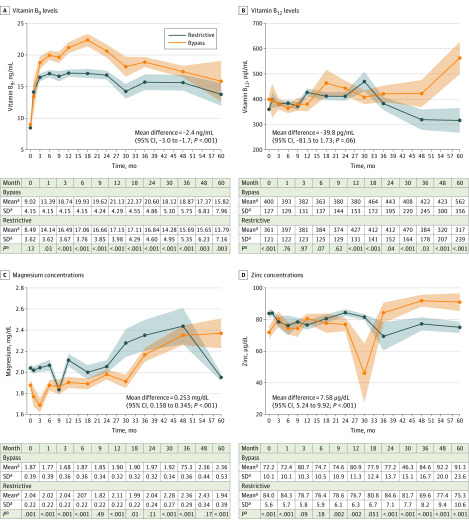
Five-year Trajectories in Serum Vitamin and Mineral Concentrations SI conversion factors: To convert vitamin B_9_ to nanomoles per liter, multiply by 2.266; B_12_ to picomoles per liter, multiply by 0.7378; and magnesium to millimoles per liter, multiply by 0.4114. ^a^Estimated margins and 95% CIs are plotted immediately after fitting inverse probability of treatment weighted linear mixed models conditioned on the treatment propensity score and additionally adjusted for sex. Restrictive includes patients who underwent laparoscopic sleeve gastrectomy; bypass includes patients who underwent laparoscopic Roux-en-Y gastric bypass or one-anastomosis gastric bypass.

Patients who underwent OAGB or LRYGB had higher serum folate (ie, vitamin B_9_) levels compared with patients who underwent LSG (mean, 2.376 [95% CI, 1.716 to 3.036] ng/mL [to convert to nanomoles per liter, multiply by 2.266]; *P* < .001) (Figure 3A; eFigure 9 in the Supplement), which likely reflects the prescription of higher dosages of folate supplements by treating physicians as a countermeasure against the relatively lower hemoglobin levels in the OAGB or LRYGB group. Supporting this hypothesis, vitamin B_12_ levels were also numerically, albeit not statistically significantly, higher among patients who underwent OAGB or LRYGB compared with those who underwent LSG (mean difference, 39.85 [95% CI, −1.73 to 81.46] pg/mL [to convert to picomoles per liter, multiply by 0.7378]; *P* = .06) (Figure 3B; eFigure 9 in the Supplement). However, patients who underwent LSG had higher mean serum concentrations of magnesium (mean difference, 0.25 [95% CI, 0.16 to 0.35] mg/dL [to convert to millimoles per liter, multiply by 0.4114]; *P* < .001) and zinc (mean difference, 7.58 [95% CI, 5.24 to 9.92] µg/dL [to convert to micromoles per liter, multiply by 0.153]; *P* < .001) during follow-up (Figure 3C and D; eFigure 10 in the Supplement).

### Global Nutrition Status

We assessed serum albumin as a marker for global nutrition status, as macronutrient deficiency could potentially mediate or exacerbate micronutrient deficiencies. A total of 595 patients contributed observations to the analyses of serum albumin (eFigure 11 in the Supplement). We did not observe any clinically significant difference in albumin levels between the LSG and OAGB or LRYGB groups that could otherwise confound subsequent comparisons of micronutrient levels between surgical procedures (mean difference, 0.06 [95% CI, −0.01 to 0.13] g/dL [to convert to grams per liter, multiply by 10]; *P* = .09). Likewise, we also compared percentage of total weight lost between LSG vs OAGB or LRYGB groups and found no difference in percentage of total weight lost during follow-up (mean difference, 0% [95% CI, −3.48% to 3.48%]; *P* > .99) (eFigure 12 in the Supplement).

Additional detailed results regarding the trajectories of all micronutrients and metabolic parameters, as well as sensitivity analyses using the raw (ie, nonimputed) data without propensity score weighting, are reported in the eAppendix in the Supplement.

## Discussion

To our knowledge, this prospective comparative effectiveness study of postbariatric procedure nutritional outcomes is the largest from Asia, among few to comprehensively interrogate the longitudinal trajectories of micronutrient levels of which deficiencies are associated with development of late metabolic complications,^12,13,14,15,16,17,18,19^ and the first to use treatment propensity scores and longitudinal mixed models to address selection and confounding biases and the time-varying nutritional changes associated with predominantly restrictive (ie, LSG) vs malabsorptive (ie, OAGB or LRYGB) bariatric procedures. The findings from this study can be regarded to reflect an audit of and to be generalizable to the contemporary real-world practice of many bariatric surgical units. In recent years, LSG has rapidly gained popularity to become the most frequently performed bariatric procedure, as is the case in our institution.^38^

Since vitamin D_2_ and elemental calcium supplements were stipulated as part of our postbariatric treatment protocol, serum 25-hydroxyvitamin D levels were unsurprisingly higher at all points compared with baseline, and there was no statistically significant difference between patients who underwent LSG compared with those who underwent OAGB or LRYGB during follow-up. Interestingly, intact parathyroid hormone levels were fairly stable in our study and stand in contrast with previous findings by Johnson and colleagues,^12^ who reported progressively increasing parathyroid hormone levels over time. An important difference is that many historical studies used a far lower dose of vitamin D (eg, 800 IU in the study by Johnson et al^12^). Therefore, we surmise that the aggressive vitamin D replacement regimen used in our study is safe yet may be more efficacious than previous studies using lower dosages for preventing increases in parathyroid hormone levels.

Anemia is the most prevalent nutritional complication of bariatric procedures reported in the literature, occurring in as many as two-thirds of patients.^19,39^ Indeed, in our study, postbariatric procedure hemoglobin levels in the overall cohort were significantly attenuated at all points and never recovered to prebariatric procedure levels. However, patients who underwent OAGB or LRYGB recorded a steeper decrease in hemoglobin during the first 3 months compared with those undergoing LSG, and hemoglobin levels remained statistically lower in the OAGB or LRYGB group than the LSG group for the rest of follow-up. These temporal trends indicate that surveillance and prophylactic efforts against anemia should commence earlier for patients undergoing OAGB or LRYGB than those undergoing LSG, preferably within the first 3 months or even before the procedure.^40,41^

This study also represents one of the first reports to delineate the interactions among sex, follow-up time, and nutritional outcomes after bariatric intervention. A novel finding is that among women, hemoglobin levels continued to decline in a strongly monotonic fashion during follow-up, whereas such a monotonically decreasing trend was not observed among men, thus indicating that nutritional support for women who underwent bariatric procedures was inadequate compared with support for men. Furthermore, hemoglobin levels were similar despite ferritin levels being consistently higher among women who underwent OAGB or LRYGB compared with LSG. This observation may reflect an unsuccessful attempt by the treating physicians to counteract the declining hemoglobin levels by prescribing iron supplements more aggressively in women undergoing OAGB or LRYGB. Recognition of such sex- and time-dependent associations of hematological complications after bariatric procedures has been largely missing from current guidelines and represents an area that warrants further investigation.

The etiological origin of postbariatric procedure anemia is complex and multifactorial: although anemia most commonly results from iron, folate, or vitamin B_12_ deficiencies, it can also manifest owing to insufficient intake of zinc, copper, and vitamins A and E.^19^ Intriguingly, although hemoglobin levels were lower in the OAGB or LRYGB group than the LSG group, this cannot be attributed to inadequate iron intake, as serum iron, total iron-binding capacity, and ferritin concentrations were comparable between groups. In fact, folate (ie, vitamin B_9_) levels were higher in the OAGB or LRYGB group than the LSG group for most of follow-up; thus, vitamin B deficiencies are unlikely to be the cause of reduced hemoglobin synthesis in our study. A 2014 meta-analysis found that malabsorptive bariatric procedures (specifically, LRYGB) were associated with greater odds of vitamin B_12_ deficiency than LSG, but we found no statistically significant difference in serum vitamin B_12_ concentrations between groups.^11^ A possible explanation for this observation is that treating physicians may have provided more intensive prescriptions to patients who underwent OAGB or LRYGB in an attempt to remedy the declining hemoglobin concentrations in among these patients compared with those who underwent LSG. Therefore, we surmise that the comparatively lower hemoglobin levels among patients who underwent OAGB or LRYGB could potentially be associated with their lower serum zinc concentrations or perhaps deficiencies in other micronutrients, such as copper and vitamins A and E, that were not collected in our study.

Micronutrient deficiencies after bariatric procedures are clinically significant,^12,13,14,15,16,17,18,19^ yet no consensus exists regarding the optimal schedule and overall length of postbariatric procedure biochemical monitoring. Furthermore, recent surveys and reviews indicate that there is significant discordance between clinical practice guidelines published by different societies regarding vitamin and trace element supplementation, and poor adherence among surgeons to any of these guidelines.^42,43,44^ Experts have articulated that the challenge with developing a set of universally accepted guidelines lies in the methodological aspects of studies performed in this field. First, the current evidence base concerning postbariatric procedure micronutrient deficiencies is largely predicated on studies that reported prevalence of micronutrient deficits and endocrine derangements at single time points. Unfortunately, single-point measurements may be unrepresentative because of the temporal fluctuations in micronutrient levels. Such temporal patterns are important to capture, as they may provide clinically pertinent insights into the rate at which reserves are depleted or replenished or reflect important pathophysiological nuances (eg, anemias associated with iron deficiency are thought to manifest earlier, whereas anemias associated with vitamin B_9_ and B_12_ deficiencies occur much later in the postoperative course^19,45^). Second, also stemming from the issue of single-point sampling, is the difficulty of synthesizing evidence in systematic reviews and meta-analyses if sampling schedules differ across studies. Third, many studies also convey the extent of nutritional deficits in terms of prevalence, which is problematic because the dichotomization of continuous parameters is associated with reduced statistical power and also inconsistencies in laboratory cutoff values for defining deficiencies.

### Strengths and Limitations

This study has some strengths. We used IPTW after fitting a propensity score model to obtain marginal contrasts of the treatment effect, which is defined as the difference in population effects if all patients in the study had been assigned to OAGB or LRYGB or to LSG. Inverse probability-of-treatment weighting is a popular method in the statistical toolbox for causal inference, as it addresses the problem of unobserved counterfactuals: since the same patient cannot possibly be assigned to both types of bariatric procedures at once, the outcome of either treatment would always be unobserved. Using IPTW overcomes this problem by creating a pseudopopulation to estimate the difference between 2 counterfactuals and estimating the treatment effects had all participants who underwent OAGB or LRYGB been assigned to LSG instead. Another analytical strength of our study is the application of generalized linear mixed-models to efficiently use all data in this longitudinal study, which allowed us to scrutinize and account for the temporal changes in serum micronutrient levels and other metabolic parameters, in contrast with many previous studies that analyzed these outcomes at single (or fewer) cross-sectional time points.

Other strengths of our study worth highlighting include this study being the largest Asian cohort to our knowledge to date, with representation by 3 major Asian ethnicities (Chinese, Malay, and Indian).^30^ This is notable because of the rapidly increasing popularity of bariatric procedures in Asia. The Asian bariatric procedure landscape has changed considerably as the epicenter of obesity and diabetes has shifted to Asia in recent years. Two of the world’s most populous and rapidly urbanizing countries, India and China, recorded the highest numbers of obese children in 2015, which could foreshadow a considerable future burden of overweight and obesity in these regions.^40,46,47,48,49,50^ Also noteworthy of this study is that it reports a larger and more comprehensive catalog of parameters than many previous studies, and especially focuses on calcium, iron, and vitamin B metabolism, as deficiencies of these micronutrients mediate some of the most prevalent metabolic complications after bariatric procedures (eg, anemia and osteoporotic fractures).

Our study also has some limitations, including the multiplicity of statistical comparisons, which is related to the large panel of biochemical parameters assessed at multiple points for up to 5 years of follow-up. An additional limitation is the nonrandomized nature of our data, which carries caveats, such as patient nonadherence, self-medication, and loss to follow-up. Nevertheless, the prospective nature of our data can also be regarded to represent the real-world contemporary experience of most bariatric procedure units. Furthermore, we also strived to minimize selection and confounding biases using IPTW.^22,23,24,25,26,27,28,29^

## Conclusions

The findings of this study suggest that choice of bariatric procedure may be associated with differences in trajectories of micronutrient levels related to erythropoiesis and bone metabolism. Our study also demonstrates that micronutrient levels after bariatric procedure are not constant, but rather, fluctuate over time, and this has implications for the interpretation of previous investigations that used a single-point measurement and the design of future studies in this developing research area. Furthermore, the findings of our subgroup analyses by sex suggest that guidelines for postbariatric procedure biochemical monitoring and nutritional management should recognize that women may be more susceptible to anemia and are likely to require more intense surveillance and prophylactic supplementation. Our results also support the utility of high-dose vitamin D_2_ supplementation and physician-directed dose modification to mitigate some of the nutritional and metabolic disturbances associated with bariatric interventions, such as increasing parathyroid hormone levels. Future research directions include examining how prebariatric procedure energy-restricted diets could entrench micronutrient deficiencies,^41^ scrutinizing whether race/ethnicity and cultural factors influence micronutrition, and more detailed longitudinal phenotyping of patients using biomarkers, such as bone mineral density and osteoclast activity.

## Notes

Supplement. **eAppendix.** Supplemental Results **eFigure 1.** Discrimination of the Propensity Score Model **eFigure 2.** Calibration and Goodness-of-Fit of the Propensity Score Model Stratified by Deciles **eFigure 3.** Temporal Course of Serum 25-Hydroxyvitamin D Concentrations and Intact Parathyroid Hormone Levels **eFigure 4.** Temporal Course of Calcium and Phosphate Concentrations **eFigure 5.** Temporal Course of Ferritin Concentrations and Hemoglobin Levels **eFigure 6.** Subgroup Analyses of Ferritin Concentrations and Hemoglobin Levels by Sex **eFigure 7.** Temporal Course of Serum Iron Concentrations and Total Iron-Binding Capacity **eFigure 8.** Subgroup Analyses of Serum Iron Concentrations and Total Iron-Binding Capacity by Sex **eFigure 9.** Temporal Course of Vitamin B_12_ and Vitamin B_9_ Levels **eFigure 10.** Temporal Course of Magnesium and Zinc Concentrations **eFigure 11.** Temporal Course of Serum Albumin Levels **eFigure 12.** Box-and-Whisker Plots for Percentage of Total Weight Lost
