# Supplementary material for: Associations of Bariatric Interventions With Micronutrient and Endocrine Disturbances
Source: JAMA Netw Open. 2020 Jun 9;3(6):e205123. doi: 10.1001/jamanetworkopen.2020.5123 (PMC7284307; doi:10.1001/jamanetworkopen.2020.5123)
Supplement: Supplement. — eAppendix. Supplemental Results eFigure 1. Discrimination of the Propensity Score Model eFigure 2. Calibration and Goodness-of-Fit of the Propensity Score Model Stratified by Deciles eFigure 3. Temporal Course of Serum 25-Hydroxyvitamin D Concentrations and Intact Parathyroid Hormone Levels eFigure 4. Temporal Course of Calcium and Phosphate Concentrations eFigure 5. Temporal Course of Ferritin Concentrations and Hemoglobin Levels eFigure 6. Subgroup Analyses of Ferritin Concentrations and Hemoglobin Levels by Sex eFigure 7. Temporal Course of Serum Iron Concentrations and Total Iron-Binding Capacity eFigure 8. Subgroup Analyses of Serum Iron Concentrations and Total Iron-Binding Capacity by Sex eFigure 9. Temporal Course of Vitamin B12 and Vitamin B9 Levels eFigure 10. Temporal Course of Magnesium and Zinc Concentrations eFigure 11. Temporal Course of Serum Albumin Levels eFigure 12. Box-and-Whisker Plots for Percentage of Total Weight Lost [file jamanetwopen-3-e205123-s001.pdf]

Supplementary Online Content

Syn NL, Lee PC, Kovalik J-P, et al. Associations of bariatric interventions with micronutrient and endocrine disturbances. *JAMA Netw Open*. 2020;3(6):e205123. doi:10.1001/jamanetworkopen.2020.5123

eAppendix. Supplemental Results

eFigure 1. Discrimination of the Propensity Score Model

eFigure 2. Calibration and Goodness-of-Fit of the Propensity Score Model Stratified by Deciles

eFigure 3. Temporal Course of Serum 25-Hydroxyvitamin D Concentrations and Intact Parathyroid Hormone Levels

eFigure 4. Temporal Course of Calcium and Phosphate Concentrations

eFigure 5. Temporal Course of Ferritin Concentrations and Hemoglobin Levels

eFigure 6. Subgroup Analyses of Ferritin Concentrations and Hemoglobin Levels by Sex

eFigure 7. Temporal Course of Serum Iron Concentrations and Total Iron-Binding Capacity

eFigure 8. Subgroup Analyses of Serum Iron Concentrations and Total Iron-Binding Capacity by Sex

eFigure 9. Temporal Course of Vitamin B_{12} and Vitamin B_9 Levels

eFigure 10. Temporal Course of Magnesium and Zinc Concentrations

eFigure 11. Temporal Course of Serum Albumin Levels

eFigure 12. Box-and-Whisker Plots for Percentage of Total Weight Lost

This supplementary material has been provided by the authors to give readers additional information about their work.

© 2020 Syn NL et al. *JAMA Network Open.*

eAppenedix. Supplemental Results

This **Supplement** provides sensitivity analyses using the raw data without multiple imputation or inverse probability of treatment-weighting based on estimated propensity scores. This section also focuses on describing in greater detail the trajectories of micronutrients and hematological or endocrine parameters, and subgroup analyses stratified by gender.

Changes in Calcium Metabolism

Vitamin D (25(OH)D)

594 patients contributed observations to the analysis of Vitamin D trajectories (**eFigure 3**). In this overall cohort, as well as in subgroup analyses of patients who underwent bypass and restrictive procedures, serum Vitamin D levels were significantly higher at all measured time points up to 5 years post-operatively as compared with baseline levels (all $P < 0.0001$) (**eFigure 3**). Comparing vitamin D levels between both groups, patients who underwent restrictive procedures recorded significantly higher Vitamin D concentrations as compared with patients who underwent bypass procedures (+3.2 ng/mL; 95% CI: 0.8 to 5.6; P=0.0096) at post-bariatric month 3 (**eFigure 3**).

Parathyroid Hormone

536 patients contributed observations to the longitudinal analysis of intact parathyroid hormone (IPTH) levels (**eFigure 3**). In the overall cohort, the only statistically significant ($P <$ 0.01) change from baseline IPTH levels (6.1±4.0 pmol/L) was seen at post-procedure 3 months (5.5 ± 3.3 pmol/L). There was no significant difference in IPTH levels between the restrictive and bypass groups at any measured time points.

© 2020 Syn NL et al. *JAMA Network Open.*

Among patients undergoing restrictive procedures, post-bariatric IPTH levels were significantly lower at 6 months (-1.00 pmol/L; 95% CI: -1.58 to -0.42; *P*=0.0007), 12 months (-0.96 pmol/L; 95% CI: -1.53 to -0.38; *P*=0.0011), and 18 months (-1.72 pmol/L; 95% CI: -3.26 to -0.18; *P*=0.0285) as compared with baseline (**eFigure 3**). For patients undergoing bypass procedures, post-bariatric IPTH were significantly higher at 36 months (+1.83 pmol/L; 95% CI: 0.39 to 3.27; *P*=0.0130), 48 months (+3.54 pmol/L; 95% CI: 0.42 to 6.66; *P*=0.0261), and 60 months (+3.77 pmol/L; 95% CI: 0.47 to 7.07; *P*=0.0251) as compared to baseline levels (**eFigure 3**).

Calcium (corrected)

590 patients contributed observations to serum calcium trajectories (**eFigure 4**). In the overall cohort, serum calcium levels remained relatively constant over time. However, in subgroup analysis, a significant increase in serum calcium (as compared with pre-bariatric levels) was delineated at 3 months post-procedure in the restrictive group (+0.07 mmol/L; 95% CI: 0.004 to 0.039; *P* = 0.0185) (**eFigure 4**). No difference between the restrictive and bypass groups was observed at any time during the 5-year follow-up.

Phosphate

582 patients contributed observations to the analysis of serum phosphate levels over a 5-year period (**eFigure 4**). In the overall cohort, serum phosphate levels were statistically higher at post-bariatric 6 and 12 months (**eFigure 4**). No difference between the restrictive and bypass groups was observed at any point in time during the 5-year follow-up.

© 2020 Syn NL et al. *JAMA Network Open.*

Changes in Iron Metabolism

Serum Iron

565 patients contributed observations to the analysis of serum iron trajectories (**eFigure 7**). In the overall cohort, iron levels decreased only in the 1st post-bariatric month, but subsequently increased above baseline levels, especially at 6 through 36 months (**eFigure 7**). In subgroup analyses, iron levels were higher at 6 through 24 months as compared with baseline in the restrictive group, but higher at 12 through 36 months as compared with baseline in the bypass group (**eFigure 7**). Iron levels peaked at 12 months and 36 months respectively in the restrictive and bypass groups, which represented an increase of +3.76 mmol/L (95% CI: 2.57 to 4.96; P<0.0001) and +6.86 mmol/L (95% CI: 1.23 to 14.5; P=0.0170) as compared with their pre-bariatric values (**eFigure 7**).

There was no difference in iron levels between the restrictive and bypass groups over the follow-up, except for the 3rd month where patients in the restrictive group recorded slightly higher iron levels than those who underwent bypass procedures (+1.60 mmol/L; 95% CI: 0.26 to 2.90; P=0.0190) (**eFigure 7**).

Amongst women, those undergoing restrictive or bypass procedures had comparable serum iron levels for the first 24 months, but diverged thereafter (**eFigure 8**). Amongst male bariatric patients, serum iron levels were statistically higher among those undergoing restrictive procedures than bypass procedures at the 1st month post-operatively, but became comparable in the subsequent months (**eFigure 8**).

Total Iron-Binding Capacity

Total iron-binding capacity (TIBC) was measured at multiple time-points for 560 patients over a period of 5 years (**eFigure 7**). In the overall cohort, TIBC was substantially lower at post-bariatric months 1, 6 through 24, and 36 and 48, as compared with baseline

© 2020 Syn NL et al. *JAMA Network Open.*

values (**eFigure 7**). TIBC values were largely comparable between the restrictive and bypass groups over the course of follow-up, except for post-bariatric month 30, where the restrictive group recorded a statistically higher average TIBC (+6.4 mmol/L; 95% CI: 0.8 to 12.1; *P*=0.0255) as compared with the bypass group (**eFigure 7**).

Amongst women, TIBC values were generally comparable between those undergoing restrictive or bypass procedures, except for post-bariatric months 1, 18, and 60, where women undergoing restrictive procedures recorded statistically higher TIBC as compared to women undergoing bypass surgeries. In the female subgroup, post-bariatric TIBC values were lower at all time points compared to baseline values (**eFigure 6**). Amongst male patients, TIBC values were statistically lower amongst those undergoing restrictive procedures compared to bypass procedures only at the 1st month post-operatively.

Ferritin

A total of 590 patients contributed longitudinal measurements to the analysis of serum ferritin trajectories over 5 years (e**Figure 5**). In the overall cohort, serum ferritin was higher at post-bariatric months 12, 18 and 60 as compared with pre-bariatric levels. Serum ferritin did not differ significantly between the restrictive and bypass group at any time point during the study.

Intriguingly and unexpectedly, within the female subgroup of patients, post-bariatric ferritin levels were statistically higher amongst those who underwent bypass compared to restrictive procedures (average fixed-effects: +31.1 ng/mL, 95% CI: 11.6 to 50.7, *P*=0.0018) over the course of follow-up (**eFigure 6**). Amongst the male subgroup, those undergoing restrictive procedures recorded higher ferritin levels between 6 through 18 months compared to male counterparts undergoing bypass procedures (**eFigure 6**).

© 2020 Syn NL et al. *JAMA Network Open.*

Hemoglobin

Haemoglobin was longitudinally assessed over 5 years in 606 patients, and levels in the overall cohort were statistically lower at all post-bariatric time points as compared with pre-bariatric levels, except for the 3rd month (e**Figure 5**).

This observation was largely recapitulated in subgroup analyses of patients who underwent restrictive and bypass procedures; the only exceptions were the 1st post-operative month in the bypass group and the 9th post-procedure month in the restrictive group wherein hemoglobin levels did not differ significantly from baseline levels (e**Figure 5**). During the initial 3 months, the bypass group recorded a steep decline in hemoglobin levels of -0.7 g/dL (95% CI: -1.0 to -0.4; *P*<0.0001) as compared with pre-surgery levels (e**Figure 5**). On the other hand, the restrictive group recorded a more tapered decrease in hemoglobin levels up till 18 months post-procedurally (-0.5 g/dL; 95% CI: -0.8 to -0.3; *P*=0.0001) (e**Figure 5**). In both the restrictive and bypass groups, trough hemoglobin levels were recorded at month 48, with a decrease of -0.8 g/dL (95% CI: -1.3 to -0.4; *P*=0.0004) and -0.9 g/dL (95% CI: -1.3 to -0.6; *P*<0.0001) respectively as compared with pre-bariatric levels.

Over the 5-year follow-up, hemoglobin levels were on average +0.6 g/dL (95% CI: 0.1 to 1.0; *P*=0.0003) higher in the restrictive group as compared with the bypass group. The difference between the restrictive and bypass groups were statistically significant at post-bariatric months 1, 3 through 18, and 36 (e**Figure 5**).

Amongst the female subgroup of patients, there was evidence of a strongly monotonic, decreasing linear trend in haemoglobin levels over the course of follow-up (*P*=0.0005). Amongst female patients, we observed that patients undergoing restrictive procedures had statistically higher post-bariatric haemoglobin levels between 3 through 18 months (**eFigure 6**). Amongst the male subgroup of patients, patients undergoing restrictive procedures had statistically higher post-bariatric haemoglobin levels at months 3-9, 18, 30, 36, and 60 months.

© 2020 Syn NL et al. *JAMA Network Open.*

A monotonically-decreasing trend in haemoglobin levels was not detected (*P*=0.7841), however, post-bariatric levels were statistically lower at all time points compared to baseline (**eFigure 6**).

Vitamin B Metabolism

Vitamin B12 (Cobalamin)

591 patients contributed observations to the time-course analysis of serum Vitamin B12 (cobalamin) levels (**eFigure 9**). In the overall cohort, post-bariatric Vitamin B12 levels were statistically higher at 12 through 36 months, and at 60 months as compared with baseline (**eFigure 9**). On average, there was no difference in serum vitamin B12 between the restrictive and bypass groups at any time point.

Vitamin B9 (Folate)

580 patients contributed observations to the 5-year longitudinal analysis of serum vitamin B9 (folate) levels (**eFigure 9**). Serum vitamin B9 levels were significantly higher at all ensuing time points after bariatric procedures as compared to baseline levels in the overall cohort (all $P < 0.0001$) (**eFigure 9**), an observation which was also recapitulated in subgroup analyses of bypass and restrictive procedures (**eFigure 9**). Over the 5-year follow up, vitamin B9 levels were on average higher in the bypass groups as compared to the restrictive groups (average fixed-effects = +7.1; 95% CI: 1.8 to 12.4; *P*=0.0001). The greatest disparity was observed at 30 months, wherein vitamin B9 levels was +13.4 nmol/L (95% CI: 7.0 to 19.9; $P<0.0001$) higher in the bypass group vs the restrictive group (**eFigure 9**).

© 2020 Syn NL et al. *JAMA Network Open.*

Zinc and Magnesium

199 and 161 patients contributed observations to the analyses of zinc and magnesium respectively, and trajectories are depicted in **eFigure 10**. In the overall cohort, magnesium concentrations remained relatively stable and only dipped significantly at 1, 30, and 36 months compared to baseline, while post-operative zinc levels did not differ significantly compared to baseline (**eFigure 10**). Serum zinc concentrations generally did not differ between the restrictive and bypass groups; however, magnesium levels were, on average, higher in the restrictive group than in the bypass group during follow-up (fixed-effects = +0.078; 95% CI: 0.035 to 0.121; *P*=0.0004).

Global nutrition status: Serum Albumin and %Total Weight Loss

We assessed serum albumin as a marker for global nutrition status, as macronutrient deficiency could potentially mediate or exacerbate micronutrient deficiencies. 595 patients contributed observations to the analyses of serum albumin (**eFigure 11**). We did not observe any clinically significant difference in albumin levels between the restrictive and bypass groups which could otherwise confound subsequent comparisons of micronutrient levels between surgical procedures (average fixed-effects = 0.6 g/L, 95% CI: -0.07 to 1.27; *P*=0.0875). Although there appeared to be a monotonically increasing trend in albumin levels over the course of follow-up, albumin levels were not statistically higher after bariatric surgery compared to baseline values (**eFigure 11**). Likewise, we also compared percentage change in total weight loss (%TWL) between LSG versus OAGB/LRYGB and found no difference in %TWL over the course of follow-up (MD = +0.00%, 95% CI: -3.48% to 3.48%; *P*=1.0000) (**eFigure 12**).

eFigure 1. Discrimination of the Propensity Score Model

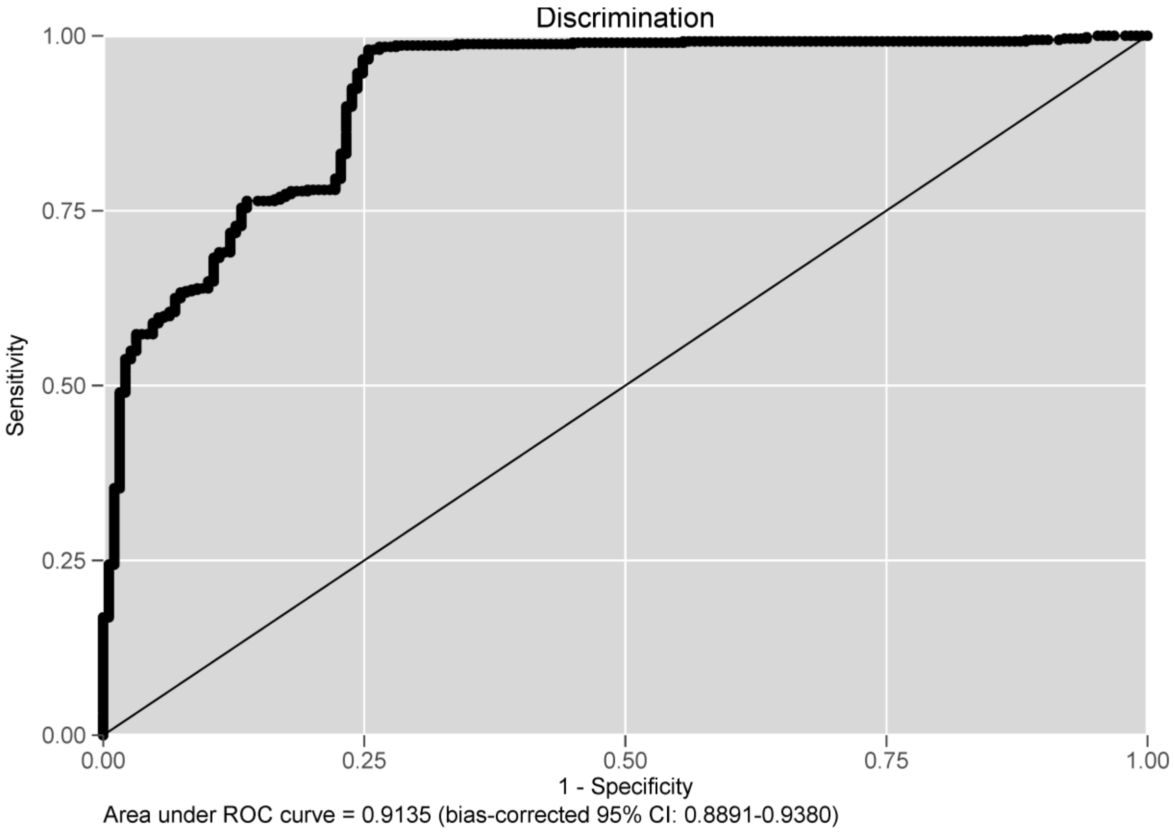

© 2020 Syn NL et al. *JAMA Network Open.*

eFigure 2. Calibration and Goodness-of-Fit of the Propensity Score Model Stratified by Deciles

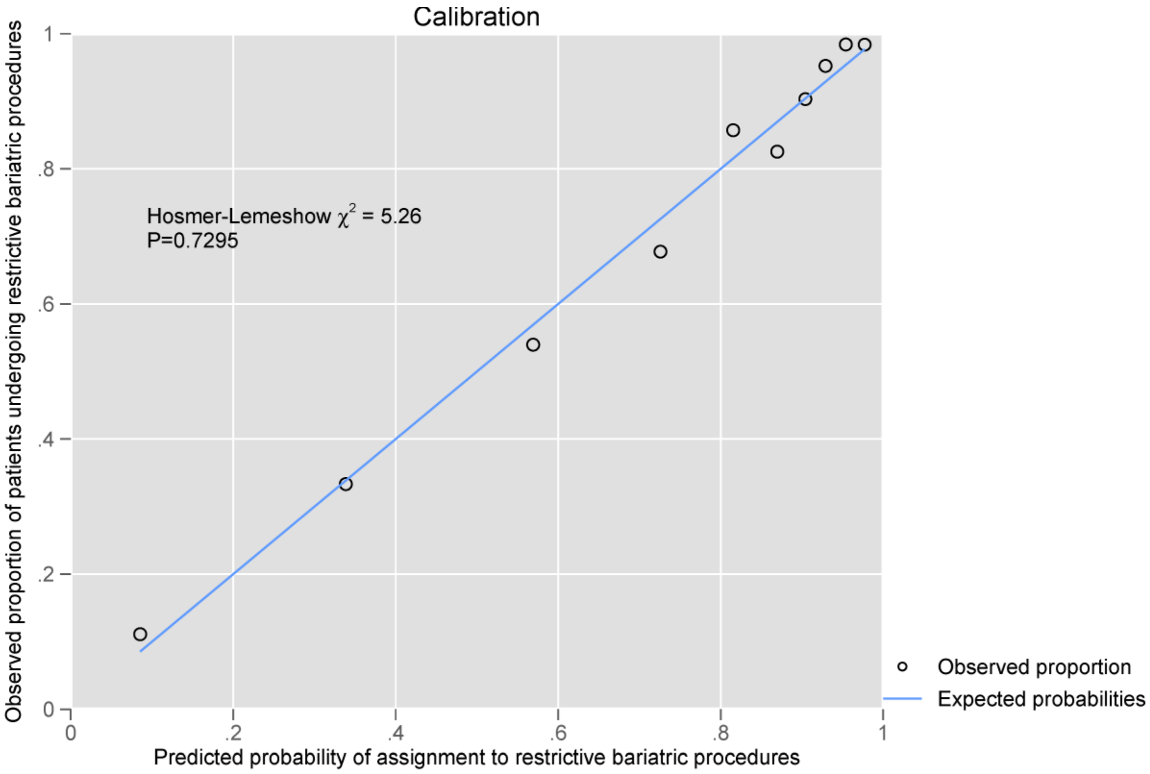

© 2020 Syn NL et al. *JAMA Network Open.*

eFigure 3. Temporal Course of Serum 25-Hydroxyvitamin D Concentrations and Intact Parathyroid Hormone Levels

The left panels represent data from the overall cohort, and include a locally-weighted least-squares running line, as well as box-and-whisker plots in which boxes were drawn to encompass the interquartile range (IQR) and whiskers drawn to span all data points within 1.5 IQR of the nearer quartile. Post-bariatric biochemical readings were compared with baseline values, and statistical significance is denoted as *, **, ***, **** for *P* < 0.05, 0.01, 0.001, and 0.0001 respectively. The tables below the left panels reflects unadjusted (raw) values. The right panels represent data from the subgroups of patients who underwent restrictive or malabsorptive procedures, and predictive margins and their standard errors were obtained after fitting longitudinal mixed-effects models.

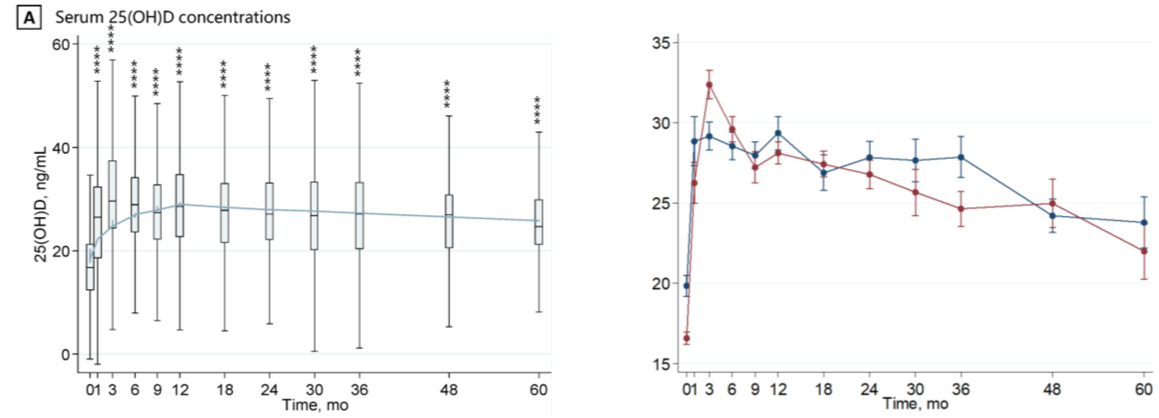

Months	0	1	3	6	9	12	18	24	30	36	48	60
Mean	17.7	26.9	31.3	29.5	27.8	29.2	27.8	28.1	27.2	27.7	26.5	25.7
SD	7.3	10.7	11.2	9.9	8.0	9.9	8.9	8.6	9.9	8.9	8.0	7.7
Median	16.7	26.5	29.7	28.9	27.4	28.6	27.8	27.1	26.8	27.1	27.0	24.7

	Months	0	1	3	6	9	12	18	24	30	36	48	60
Bypass	Mean	19.8	28.8	29.2	28.6	28.0	29.4	26.9	27.8	27.7	27.9	24.2	23.8
	SE	.65	1.54	.86	.84	.82	1.04	1.10	1.01	1.33	1.27	1.04	1.59
Restrictive	Mean	16.6	26.3	32.4	29.6	27.2	28.1	27.4	27.8	25.7	24.6	25.0	22.0
	SE	.37	1.27	.89	.79	.97	.70	.81	.89	1.44	1.09	1.51	1.72

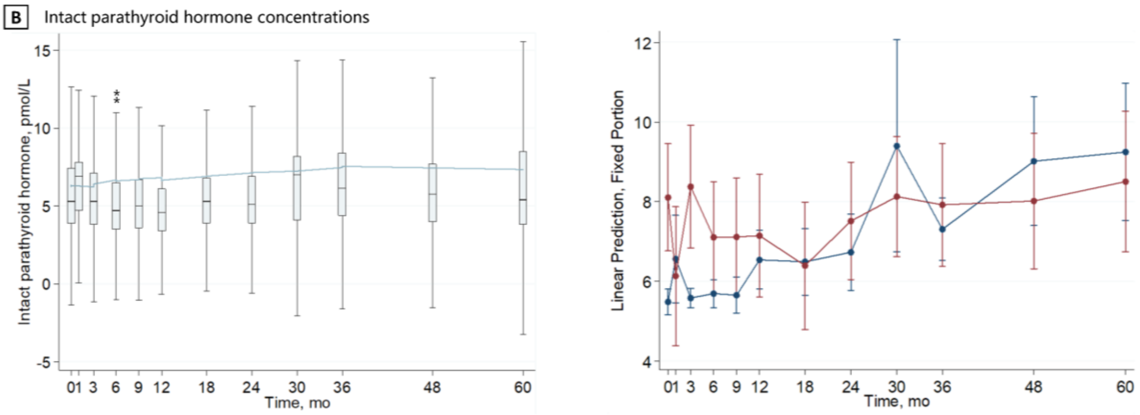

Months	0	1	3	6	9	12	18	24	30	36	48	60
Mean	6.1	8.6	6.5	5.5	7.9	7.9	6.3	6.2	8.6	7.1	8.6	6.2
SD	4.0	9.2	8.1	3.3	12.1	33.7	5.5	6.4	14.7	6.4	15.4	3.0
Median	5.3	6.9	5.3	4.7	5.0	4.6	5.3	5.1	7.0	6.2	5.8	5.4

	Months	0	1	3	6	9	12	18	24	30	36	48	60
Bypass	Mean	5.5	6.6	5.6	5.7	5.7	6.5	6.5	6.7	9.4	7.3	9.0	9.3
	SE	.32	1.10	.24	.35	.45	.74	.83	.96	2.67	.78	1.62	1.72
Restrictive	Mean	8.1	6.1	8.4	7.1	7.1	7.1	6.4	7.5	8.1	7.9	8.0	8.5
	SE	1.35	1.74	1.54	1.40	1.49	1.54	1.60	1.47	1.51	1.55	1.70	1.77

© 2020 Syn NL et al. *JAMA Network Open.*

eFigure 4. Temporal Course of Calcium and Phosphate Concentrations

The left panels represent data from the overall cohort, and include a locally-weighted least-squares running line, as well as box-and-whisker plots in which boxes were drawn to encompass the interquartile range (IQR) and whiskers drawn to span all data points within 1.5 IQR of the nearer quartile. Post-bariatric biochemical readings were compared with baseline values, and statistical significance is denoted as *, **, ***, **** for $P < 0.05$, 0.01, 0.001, and 0.0001 respectively. The tables below the left panels reflects unadjusted (raw) values. The right panels represent data from the subgroups of patients who underwent restrictive or malabsorptive procedures, and predictive margins and their standard errors were obtained after fitting longitudinal mixed-effects models.

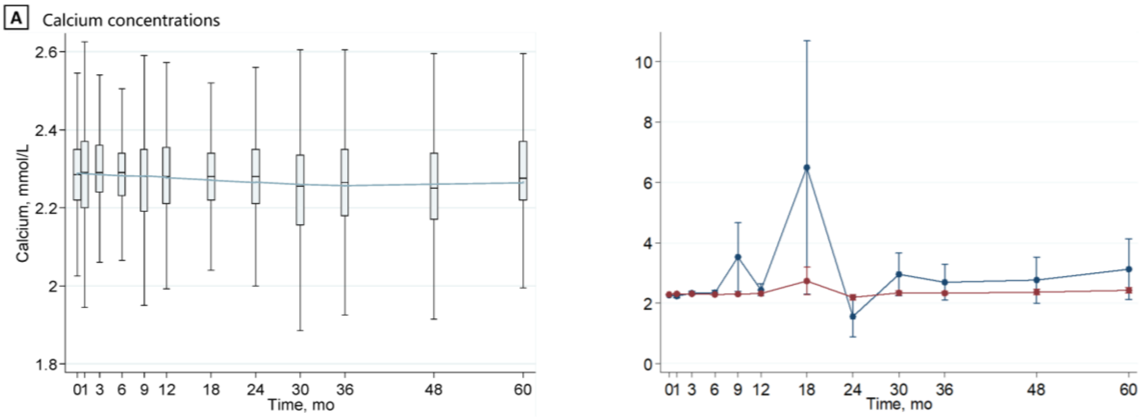

Months	0	1	3	6	9	12	18	24	30	36	48	60
Mean	2.28	2.29	2.30	2.28	2.25	2.28	2.26	2.28	2.23	2.26	2.25	2.28
SD	.10	.15	.09	.09	.21	.12	.11	.10	.16	.12	.13	.11
Median	2.29	2.29	2.29	2.29	2.28	2.28	2.28	2.28	2.23	2.27	2.25	2.28

	Months	0	1	3	6	9	12	18	24	30	36	48	60
Bypass	Mean	2.27	2.23	2.34	2.34	3.53	2.44	6.50	1.56	2.95	2.70	2.77	3.12
	SE	.009	.045	.055	.083	1.13	.198	4.19	.666	.701	.591	.767	1.00
Restrictive	Mean	2.29	2.32	2.31	2.29	2.30	2.32	2.73	2.20	2.34	2.34	2.37	2.43
	SE	.005	.022	.009	.011	.021	.021	.453	.095	.074	.053	.091	.082

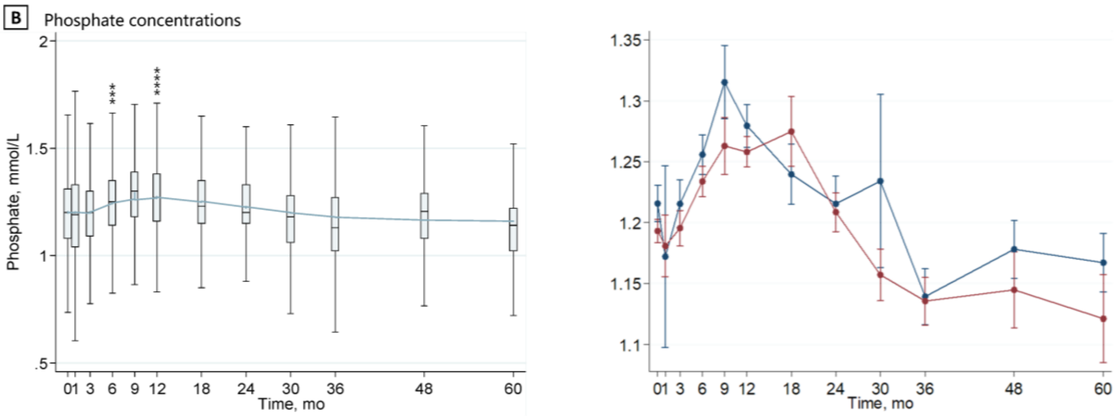

Months	0	1	3	6	9	12	18	24	30	36	48	60
Mean	1.20	1.19	1.20	1.24	1.29	1.27	1.26	1.22	1.21	1.14	1.19	1.15
SD	.18	.21	.16	.15	.19	.16	.21	.16	.30	.17	.17	.14
Median	1.20	1.19	1.20	1.25	1.30	1.27	1.23	1.20	1.18	1.13	1.21	1.14

	Months	0	1	3	6	9	12	18	24	30	36	48	60
Bypass	Mean	1.22	1.17	1.22	1.26	1.32	1.28	1.24	1.22	1.23	1.14	1.18	1.17
	SE	.015	.074	.020	.016	.030	.017	.025	.023	.071	.023	.024	.024
Restrictive	Mean	1.19	1.81	1.20	1.23	1.26	1.26	1.27	1.21	1.16	1.14	1.15	1.12
	SE	.010	.025	.015	.013	.023	.013	.029	.016	.021	.019	.031	.036

© 2020 Syn NL et al. *JAMA Network Open.*

eFigure 5. Temporal Course of Ferritin Concentrations and Hemoglobin Levels

The left panels represent data from the overall cohort, and include a locally-weighted least-squares running line, as well as box-and-whisker plots in which boxes were drawn to encompass the interquartile range (IQR) and whiskers drawn to span all data points within 1.5 IQR of the nearer quartile. Post-bariatric biochemical readings were compared with baseline values, and statistical significance is denoted as *, **, ***, **** for $P < 0.05$, 0.01, 0.001, and 0.0001 respectively. The tables below the left panels reflects unadjusted (raw) values. The right panels represent data from the subgroups of patients who underwent restrictive or malabsorptive procedures, and predictive margins and their standard errors were obtained after fitting longitudinal mixed-effects models.

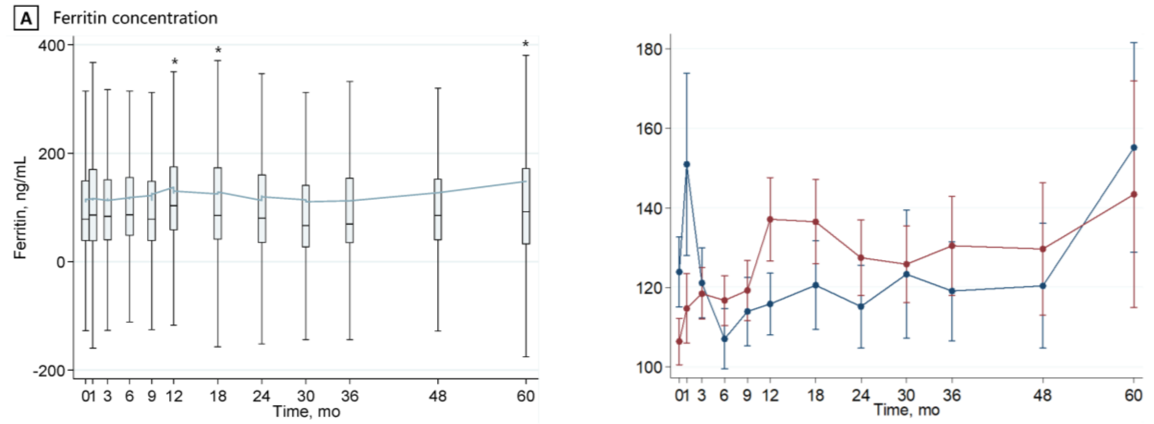

Months	0	1	3	6	9	12	18	24	30	36	48	60
Mean	111	116	113	119	114	137	128	112	101	114	119	155
SD	106	110	103	112	108	130	140	115	116	128	128	192
Median	78	85	83	86	79	103	85	80	66	69	85	92

	Months	0	1	3	6	9	12	18	24	30	36	48	60
Bypass	Mean	124	151	121	107	114	116	121	115	123	119	120	155
	SE	8.8	22.9	8.8	7.6	8.6	7.8	11.1	10.4	16.1	12.5	15.7	26.3
Restrictive	Mean	106	115	118	116	119	137	137	127	126	130	130	143
	SE	5.8	8.8	6.5	6.3	7.6	10.5	10.6	9.5	9.7	12.5	16.7	28.5

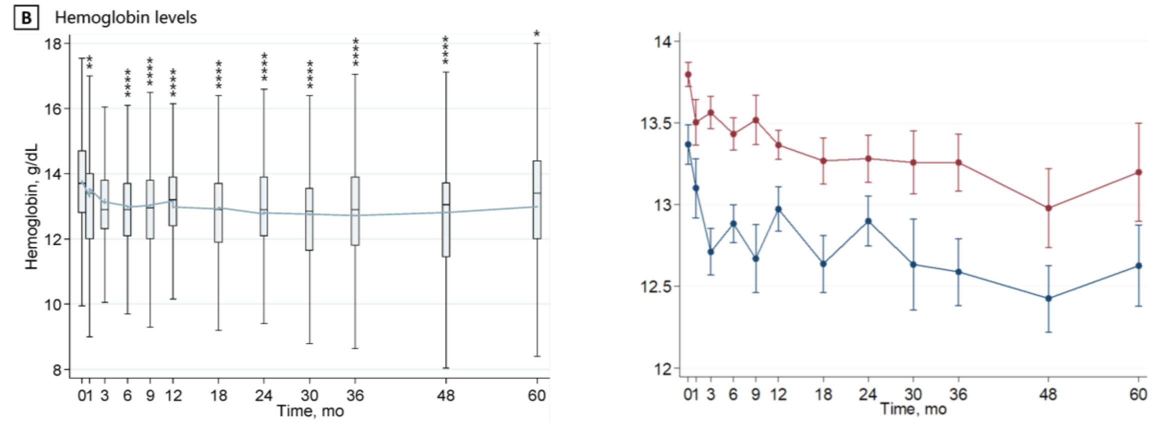

Months	0	1	3	6	9	12	18	24	30	36	48	60
Mean	13.7	13.3	13.1	13.0	12.8	13.2	12.7	12.9	12.5	12.8	12.6	13.1
SD	1.5	1.6	1.3	1.4	1.7	1.4	1.6	1.7	1.9	1.8	1.6	1.6
Median	13.7	13.4	12.9	12.9	13.0	13.2	12.9	12.9	12.9	12.9	13.1	13.4

	Months	0	1	3	6	9	12	18	24	30	36	48	60
Bypass	Mean	13.4	13.1	12.7	12.9	12.7	13.0	12.6	12.9	12.6	12.6	12.4	12.6
	SE	.12	.18	.14	.11	.21	.14	.18	.15	.28	.21	.20	.25
Restrictive	Mean	13.8	13.5	13.6	13.4	13.5	13.4	13.3	13.3	13.3	13.3	13.0	13.2
	SE	.08	.14	.10	.10	.15	.09	.14	.15	.19	.17	.24	.30

© 2020 Syn NL et al. *JAMA Network Open.*

eFigure 6. Subgroup Analyses of Ferritin Concentrations and Hemoglobin Levels by Sex

The right panels represent data from patients who underwent restrictive or malabsorptive procedures, and predictive margins and their standard errors were obtained after fitting longitudinal mixed-effects models.

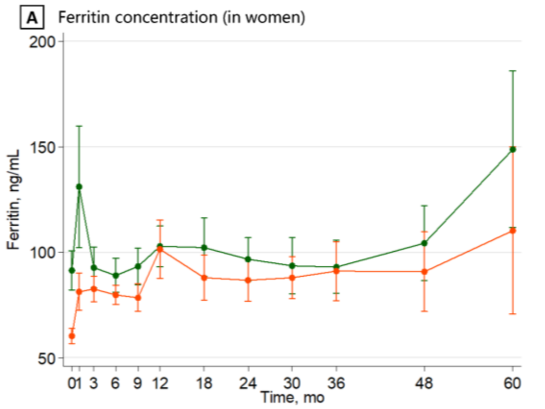

	Months	0	1	3	6	9	12	18	24	30	36	48	60
Bypass	Mean	91	131	93	89	93	103	102	97	94	93	104	149
	SE	9.3	28.9	9.7	8.1	8.8	9.7	13.8	10.3	13.4	12.6	17.6	37.1
Restrictive	Mean	60	81	83	80	79	102	88	87	88	91	91	110
	SE	3.6	8.8	6.0	4.6	6.5	13.9	10.7	9.9	9.9	14.0	18.8	39.6

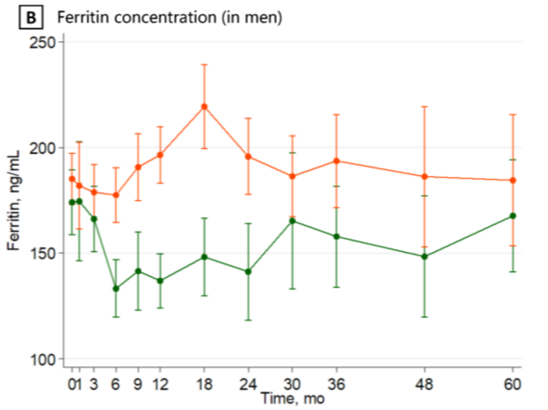

	Months	0	1	3	6	9	12	18	24	30	36	48	60
Bypass	Mean	174	175	166	133	142	137	148	141	165	158	148	168
	SE	15.3	28.1	15.5	13.6	18.5	12.8	18.3	22.9	32.2	23.9	28.7	26.6
Restrictive	Mean	185	182	179	177	191	196	219	196	186	194	186	185
	SE	12.1	20.4	13.1	13.1	15.9	13.3	19.9	18.0	19.1	22.0	33.2	31.1

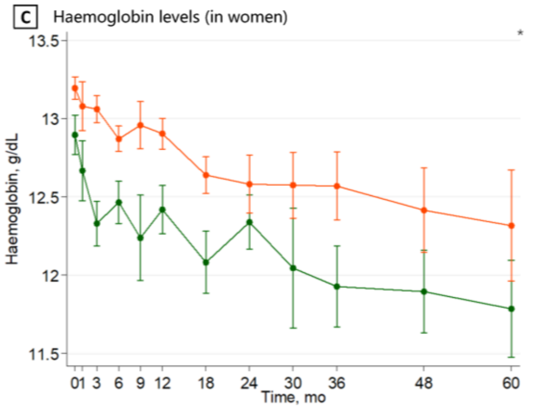

	Months	0	1	3	6	9	12	18	24	30	36	48	60
Bypass	Mean	12.9	12.7	12.3	12.5	12.2	12.4	12.1	12.3	12.0	11.9	11.9	11.8
	SE	.13	.19	.14	.13	.27	.15	.29	.17	.38	.26	.26	.31
Restrictive	Mean	13.2	13.1	13.1	12.9	13.0	12.9	12.6	12.6	12.6	12.6	12.4	12.3
	SE	.07	.16	.09	.08	.15	.10	.12	.18	.21	.22	.27	.36

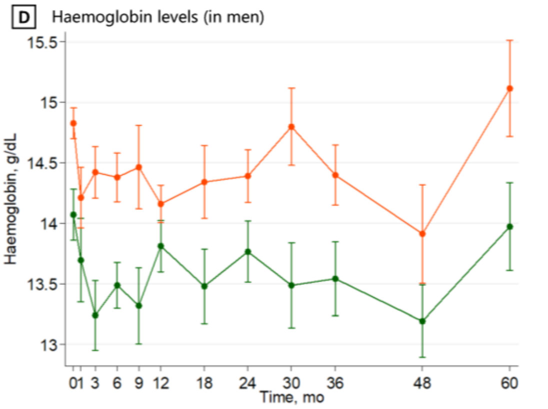

	Months	0	1	3	6	9	12	18	24	30	36	48	60
Bypass	Mean	14.1	13.7	13.2	13.5	13.3	13.8	13.5	13.8	13.5	13.5	13.2	14.0
	SE	.21	.35	.29	.19	.31	.21	.31	.25	.35	.31	.30	.36
Restrictive	Mean	14.8	14.2	14.4	14.4	14.5	14.2	14.3	14.4	14.8	14.4	13.9	15.1
	SE	.13	.25	.21	.20	.35	.15	.30	.22	.32	.25	.41	.40

© 2020 Syn NL et al. *JAMA Network Open.*

eFigure 7. Temporal Course of Serum Iron Concentrations and Total Iron-Binding Capacity

The left panels represent data from the overall cohort, and include a locally-weighted least-squares running line, as well as box-and-whisker plots in which boxes were drawn to encompass the interquartile range (IQR) and whiskers drawn to span all data points within 1.5 IQR of the nearer quartile. Post-bariatric biochemical readings were compared with baseline values, and statistical significance is denoted as *, **, ***, **** for *P* < 0.05, 0.01, 0.001, and 0.0001 respectively. The tables below the left panels reflects unadjusted (raw) values. The right panels represent data from the subgroups of patients who underwent restrictive or malabsorptive procedures, and predictive margins and their standard errors were obtained after fitting longitudinal mixed-effects models.

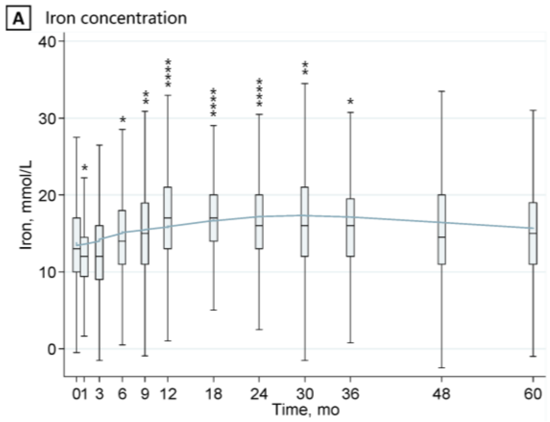

Months	0	1	3	6	9	12	18	24	30	36	48	60
Mean	13.8	12.2	13.1	15.0	15.5	17.8	17.6	17.0	16.0	18.6	16.3	14.9
SD	7.6	3.7	5.1	6.8	5.8	7.6	9.2	6.8	6.1	15.6	10.5	5.3
Median	13	12	12	14	15	17	17	16	16	16	14.5	15

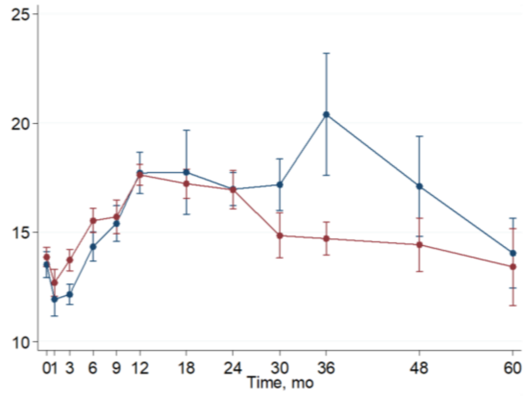

	Months	0	1	3	6	9	12	18	24	30	36	48	60
Bypass	Mean	13.5	11.9	12.2	14.3	15.4	17.7	17.8	17.0	17.2	20.4	17.1	14.0
	SE	.59	.78	.46	.66	.82	.95	1.92	.76	1.18	2.79	2.29	1.60
Restrictive	Mean	13.9	12.7	13.7	15.5	15.7	17.7	17.2	17.0	14.9	14.7	14.4	13.4
	SE	.43	.61	.49	.55	.77	.49	.67	.89	1.03	.76	1.23	1.76

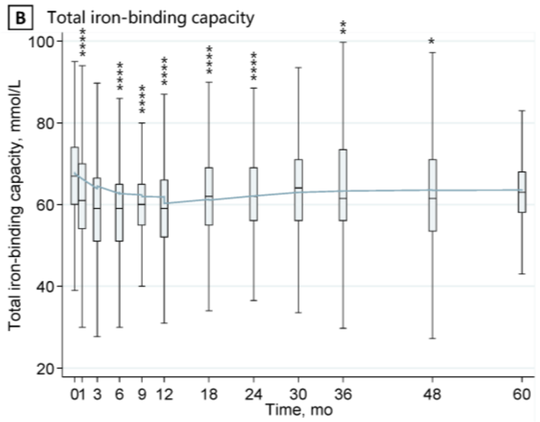

Months	0	1	3	6	9	12	18	24	30	36	48	60
Mean	67.8	60.8	65.5	58.7	60.5	59.6	61.9	62.4	65.7	63.0	63.0	63.6
SD	11.3	11.9	14.3	11.0	10.8	11.7	12.3	11.9	13.7	15.4	14.8	13.5
Median	67	61	59	59	60	59	62	62	64	61.5	61.5	63.0

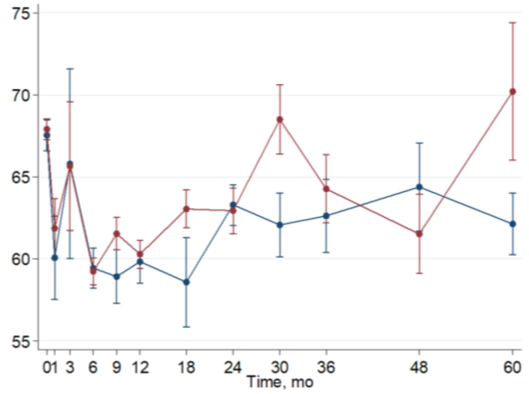

	Months	0	1	3	6	9	12	18	24	30	36	48	60
Bypass	Mean	67.5	60.1	65.8	59.4	58.9	59.8	58.6	63.3	62.1	62.6	64.4	62.1
	SE	.95	2.54	5.79	1.22	1.63	1.32	2.72	1.23	1.95	2.22	2.69	1.89
Restrictive	Mean	67.9	61.9	65.7	59.2	61.5	60.3	63.0	62.9	68.5	64.3	61.5	70.2
	SE	.64	1.82	3.93	.82	.98	.85	1.16	1.40	2.13	2.08	2.40	4.18

© 2020 Syn NL et al. *JAMA Network Open.*

eFigure 8. Subgroup Analyses of Serum Iron Concentrations and Total Iron-Binding Capacity by Sex

The right panels represent data from patients who underwent restrictive or malabsorptive procedures, and predictive margins and their standard errors were obtained after fitting longitudinal mixed-effects models.

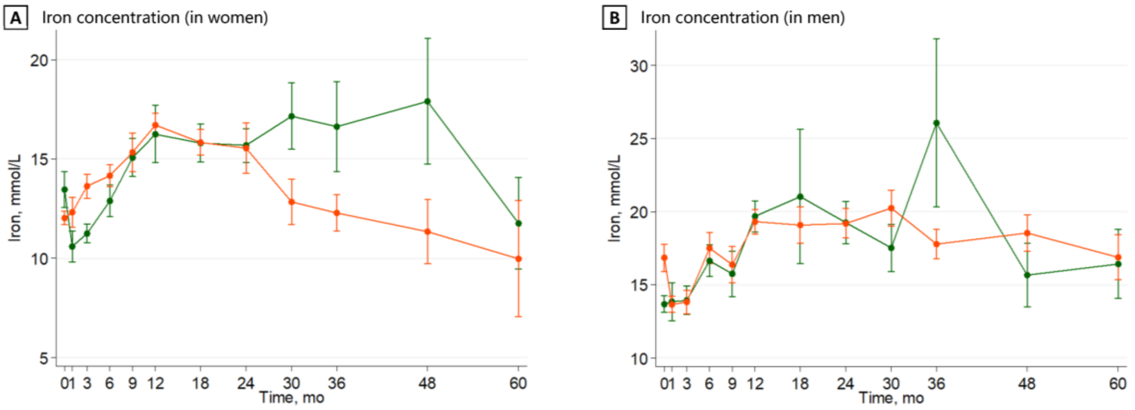

	Months	0	1	3	6	9	12	18	24	30	36	48	60
Bypass	Mean	13.5	10.6	11.3	12.9	15.1	16.3	15.8	15.7	17.2	16.6	17.9	11.8
	SE	.91	.77	.48	.80	.96	1.44	.96	.85	1.66	2.26	3.16	2.39
Restrictive	Mean	12.0	12.3	13.6	14.2	15.3	16.7	15.8	15.6	12.8	12.3	11.3	10.0
	SE	.33	.76	.62	.54	.98	.58	.64	1.27	1.13	.92	1.63	2.92

	Months	0	1	3	6	9	12	18	24	30	36	48	60
Bypass	Mean	13.7	13.8	13.9	16.6	15.8	19.7	21.0	19.3	17.5	26.1	15.7	16.4
	SE	.58	1.31	.96	1.07	1.56	1.07	4.60	1.23	1.95	2.22	2.69	1.89
Restrictive	Mean	16.9	13.7	13.8	17.5	16.4	19.3	19.1	19.2	20.2	17.8	18.5	16.9
	SE	.94	.55	.79	1.05	1.24	.86	1.25	1.01	1.23	.99	1.25	1.54

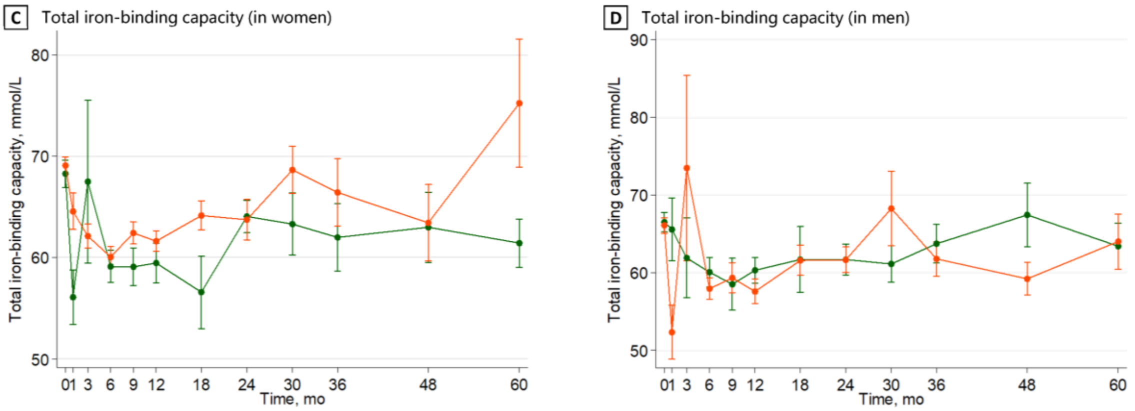

	Months	0	1	3	6	9	12	18	24	30	36	48	60
Bypass	Mean	68.3	56.1	67.5	59.1	59.1	59.4	56.6	64.0	63.3	62.0	63.0	61.4
	SE	1.35	2.66	8.05	1.59	1.86	1.96	3.54	1.59	3.08	3.31	3.47	2.38
Restrictive	Mean	69.1	64.6	62.1	60.1	62.4	61.6	64.1	63.7	68.6	66.4	63.4	75.2
	SE	.82	1.80	1.18	1.01	1.08	1.03	1.42	2.00	2.34	3.34	3.77	6.31

	Months	0	1	3	6	9	12	18	24	30	36	48	60
Bypass	Mean	66.5	65.6	61.9	60.1	58.5	60.3	61.7	61.7	61.1	63.7	67.4	63.4
	SE	1.27	4.05	5.13	1.89	3.33	1.66	4.22	2.00	2.33	2.49	4.10	2.99
Restrictive	Mean	66.1	52.3	73.5	57.9	59.3	57.6	61.6	61.6	68.3	61.7	59.2	64.0
	SE	1.00	3.47	11.95	1.38	1.95	1.56	1.94	1.64	4.81	2.24	2.09	3.53

© 2020 Syn NL et al. *JAMA Network Open.*

eFigure 9. Temporal Course of Vitamin B_{12} and Vitamin B_9 Levels

The left panels represent data from the overall cohort, and include a locally-weighted least-squares running line, as well as box-and-whisker plots in which boxes were drawn to encompass the interquartile range (IQR) and whiskers drawn to span all data points within 1.5 IQR of the nearer quartile. Post-bariatric biochemical readings were compared with baseline values, and statistical significance is denoted as *, **, ***, **** for $P < 0.05$, 0.01, 0.001, and 0.0001 respectively. The tables below the left panels reflects unadjusted (raw) values. The right panels represent data from the subgroups of patients who underwent restrictive or malabsorptive procedures, and predictive margins and their standard errors were obtained after fitting longitudinal mixed-effects models.

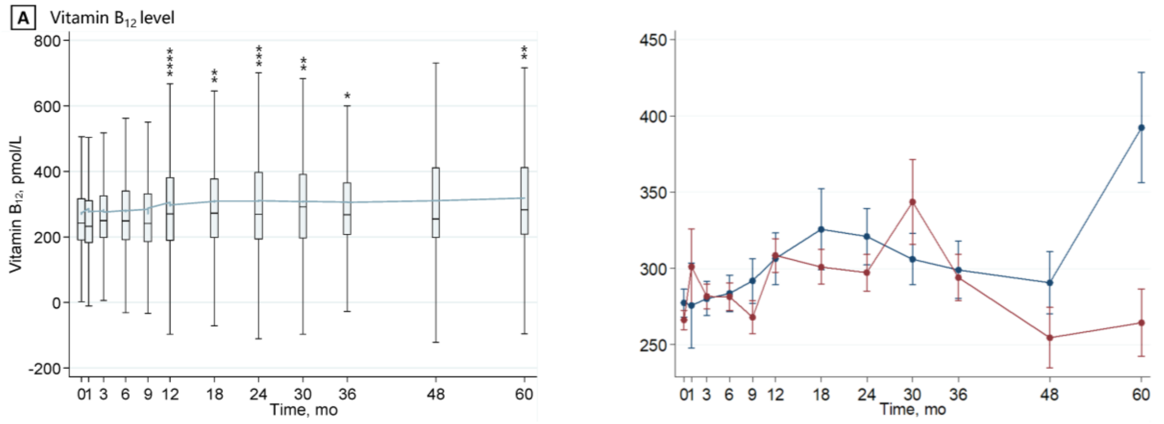

Months	0	1	3	6	9	12	18	24	30	36	48	60
Mean	269	287	279	280	271	306	313	308	312	301	312	318
SD	116	179	125	133	125	160	185	152	157	146	166	149
Median	242	232	249	248	241	270	272	269	292	268	254	283

	Months	0	1	3	6	9	12	18	24	30	36	48	60
Bypass	Mean	277	276	280	284	292	306	326	321	306	299	290	392
	SE	9.0	27.7	11.3	11.8	14.6	17.0	26.6	18.5	16.7	18.8	20.3	36.0
Restrictive	Mean	266	301	282	282	268	309	300	297	344	294	255	264
	SE	6.3	24.8	8.3	8.0	10.8	11.1	11.4	12.0	27.9	15.1	19.9	22.0

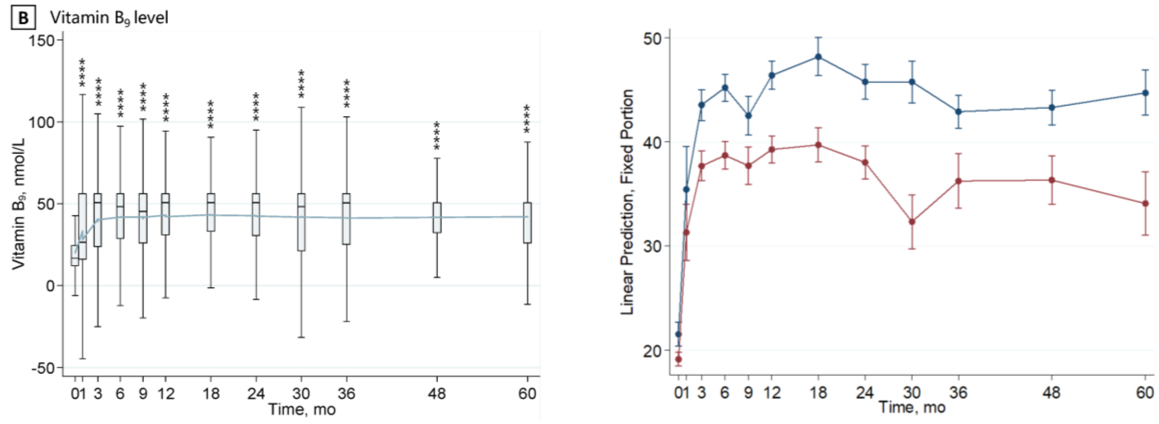

Months	0	1	3	6	9	12	18	24	30	36	48	60
Mean	20.1	33.4	40.7	41.9	40.9	43.0	44.0	43.2	39.5	40.4	42.8	41.3
SD	11.6	18.2	17.0	16.5	15.7	16.2	15.4	16.2	16.9	16.2	14.8	16.1
Median	16.6	26.3	50.5	48.2	45.3	50.5	50.5	50.5	48.2	50.4	50.5	50.5

	Months	0	1	3	6	9	12	18	24	30	36	48	60
Bypass	Mean	21.5	35.4	43.6	45.2	42.5	46.4	48.2	45.8	45.8	42.9	43.3	44.7
	SE	1.13	4.16	1.48	1.30	1.89	1.35	1.82	1.68	2.03	1.59	1.66	2.17
Restrictive	Mean	19.1	31.3	37.7	38.7	37.7	39.3	39.7	38.0	32.3	36.3	36.3	34.1
	SE	.66	2.72	1.42	1.31	1.80	1.29	1.66	1.60	2.61	2.64	2.32	3.06

© 2020 Syn NL et al. *JAMA Network Open.*

eFigure 10. Temporal Course of Magnesium and Zinc Concentrations

The left panels represent data from the overall cohort, and include a locally-weighted least-squares running line, as well as box-and-whisker plots in which boxes were drawn to encompass the interquartile range (IQR) and whiskers drawn to span all data points within 1.5 IQR of the nearer quartile. Post-bariatric biochemical readings were compared with baseline values, and statistical significance is denoted as *, **, ***, **** for *P* < 0.05, 0.01, 0.001, and 0.0001 respectively. The tables below the left panels reflects unadjusted (raw) values. The right panels represent data from the subgroups of patients who underwent restrictive or malabsorptive procedures, and predictive margins and their standard errors were obtained after fitting longitudinal mixed-effects models.

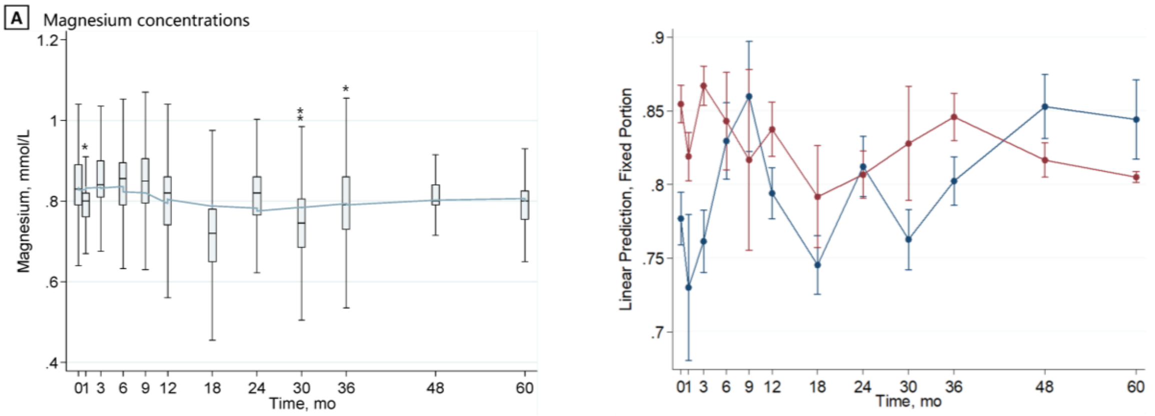

Months	0	1	3	6	9	12	18	24	30	36	48	60
Mean	.833	.793	.841	.829	.844	.801	.707	.812	.743	.790	.818	.795
SD	.086	.065	.084	.092	.153	.093	.080	.103	.088	.078	.070	.068
Median	.83	.80	.84	.86	.85	.82	.72	.82	.75	.79	.80	.80

	Months	0	1	3	6	9	12	18	24	30	36	48	60
Bypass	Mean	.777	.730	.761	.830	.860	.794	.745	.812	.763	.802	.853	.844
	SE	.018	.050	.021	.026	.037	.017	.020	.020	.020	.017	.022	.027
Restrictive	Mean	.855	.819	.867	.843	.817	.837	.792	.807	.828	.846	.817	.805
	SE	.013	.016	.013	.033	.061	.018	.035	.016	.039	.016	.012	.004

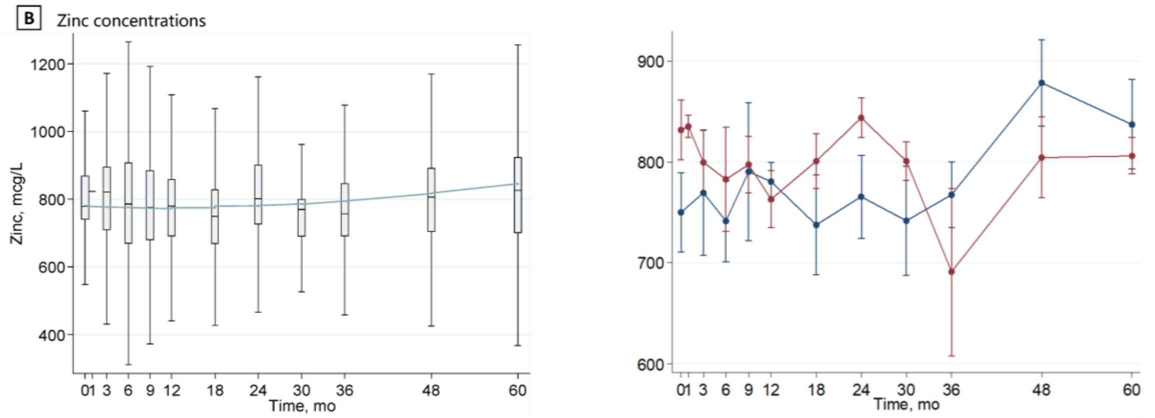

Months	0	1	3	6	9	12	18	24	30	36	48	60
Mean	785	-	767	764	797	774	757	803	759	751	861	812
SD	173	-	246	234	213	161	232	217	227	207	235	146
Median	779	-	821	785	776	780	749	801	770	757	806	826

	Months	0	1	3	6	9	12	18	24	30	36	48	60
Bypass	Mean	750	-	769	741	790	781	738	766	742	767	878	837
	SE	39.3	-	61.6	40.2	68.4	18.9	49.4	41.2	54.2	32.7	42.7	44.4
Restrictive	Mean	832	835	799	783	797	763	801	844	801	691	804	806
	SE	29.4	11.1	32.4	51.5	28.0	28.5	27.1	19.9	19.1	82.8	40.3	17.9

© 2020 Syn NL et al. *JAMA Network Open.*

eFigure 11. Temporal Course of Serum Albumin Levels

A running line was drawn using locally-weighted least-squares regression. The left panels represent data from the overall cohort, and include a locally-weighted least-squares running line, as well as box-and-whisker plots in which boxes were drawn to encompass the interquartile range (IQR) and whiskers drawn to span all data points within 1.5 IQR of the nearer quartile. Post-bariatric biochemical readings were compared with baseline values, and statistical significance is denoted as *, **, ***, **** for *P* < 0.05, 0.01, 0.001, and 0.0001 respectively. The tables below the left panels reflects unadjusted (raw) values. The right panels represent data from the subgroups of patients who underwent restrictive or malabsorptive procedures, and predictive margins and their standard errors were obtained after fitting longitudinal mixed-effects models.

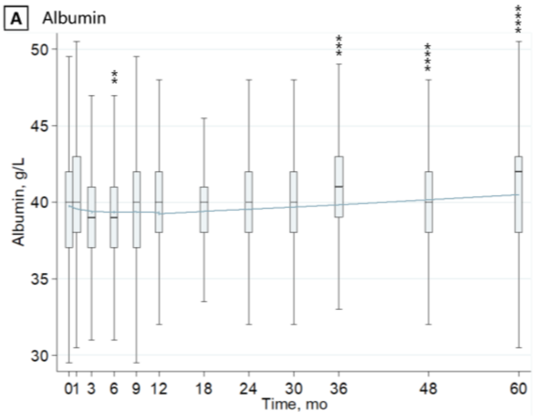

Months	0	1	3	6	9	12	18	24	30	36	48	60
Mean	39.7	40.1	39.0	38.8	39.2	39.4	39.0	39.7	39.3	40.1	39.9	40.7
SD	3.5	4.1	3.5	3.9	5.3	4.2	4.5	3.4	4.2	3.8	3.7	3.8
Median	40	40	39	39	40	40	40	40	40	41	40	42

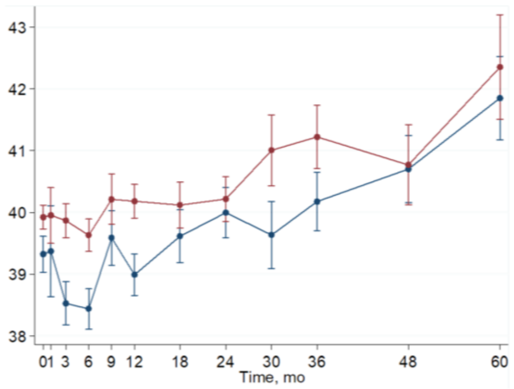

	Months	0	1	3	6	9	12	18	24	30	36	48	60
Bypass	Mean	39.3	39.4	38.5	38.4	39.6	39.0	39.6	40.0	39.6	40.2	40.7	41.9
	SE	.29	.74	.35	.33	.45	.34	.43	.40	.55	.47	.54	.67
Restrictive	Mean	39.9	40.0	39.9	39.6	40.2	40.2	40.1	40.2	41.0	41.2	40.8	42.4
	SE	.20	.45	.28	.26	.41	.27	.37	.37	.57	.51	.65	.85

© 2020 Syn NL et al. *JAMA Network Open.*

eFigure 12. Box-and-Whisker Plots for Percentage of Total Weight Lost

boxes were drawn to encompass the interquartile range (IQR) and whiskers drawn to span all data points within 1.5 IQR of the nearer quartile.

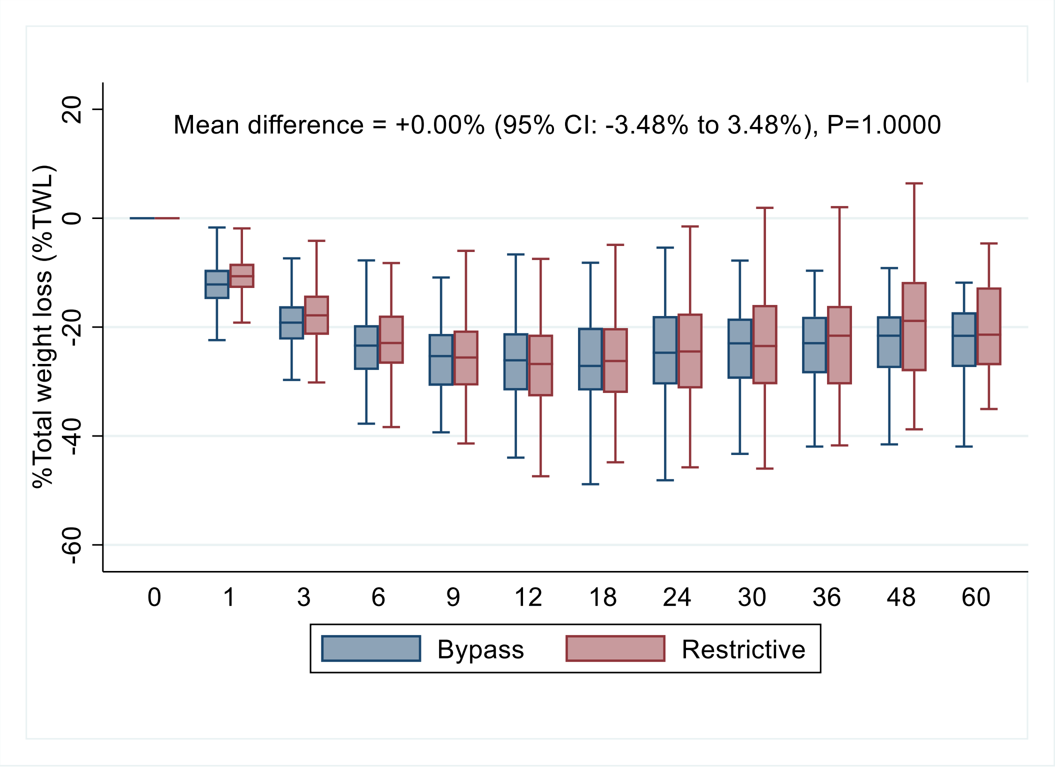

© 2020 Syn NL et al. *JAMA Network Open.*
